# Optogenetic Stimulation of Prelimbic Pyramidal Neurons Maintains Fear Memories and Modulates Amygdala Pyramidal Neuron Transcriptome

**DOI:** 10.3390/ijms22020810

**Published:** 2021-01-15

**Authors:** Daniela Laricchiuta, Giuseppe Sciamanna, Juliette Gimenez, Andrea Termine, Carlo Fabrizio, Silvia Caioli, Francesca Balsamo, Anna Panuccio, Marco De Bardi, Luana Saba, Noemi Passarello, Debora Cutuli, Anna Mattioni, Cristina Zona, Valerio Orlando, Laura Petrosini

**Affiliations:** 1 Department of Experimental Neuroscience, IRCCS Fondazione Santa Lucia, 00143 Rome, Italy; g.sciamanna@hsantalucia.it (G.S.); j.gimenez@hsantalucia.it (J.G.); andreatermine544@gmail.com (A.T.); carlo.fabrizio217@gmail.com (C.F.); francesca.balsamo93@gmail.com (F.B.); anna.panuccio@gmail.com (A.P.); m.debardi@hsantalucia.it (M.D.B.); luana_saba@libero.it (L.S.); noemi.passarello@gmail.com (N.P.); debora_cutuli@yahoo.it (D.C.); Anna.Mattioni@gmail.com (A.M.); valerio.orlando@kaust.edu.sa (V.O.); laura.petrosini@uniroma1.it (L.P.); 2 Department of Systems Medicine, Tor Vergata University of Rome, 00133 Rome, Italy; zona@uniroma2.it; 3 Unit of Neurology, IRCCS Neuromed, 86077 Pozzilli, Italy; silviacaioli@yahoo.it; 4 Department of Psychology, University “Sapienza” of Rome, 00185 Rome, Italy; 5 Biological Environmental Science and Engineering Division, KAUST Environmental Epigenetics Program, Thuwal 23955-6900, Saudi Arabia

**Keywords:** fear extinction, fear conditioning, medial prefrontal cortex, RNA sequencing, differential gene expression, electrophysiological recordings, excitatory post-synaptic currents, spinogenesis, fear-related disorders

## Abstract

Fear extinction requires coordinated neural activity within the amygdala and medial prefrontal cortex (mPFC). Any behavior has a transcriptomic signature that is modified by environmental experiences, and specific genes are involved in functional plasticity and synaptic wiring during fear extinction. Here, we investigated the effects of optogenetic manipulations of prelimbic (PrL) pyramidal neurons and amygdala gene expression to analyze the specific transcriptional pathways associated to adaptive and maladaptive fear extinction. To this aim, transgenic mice were (or not) fear-conditioned and during the extinction phase they received optogenetic (or sham) stimulations over photo-activable PrL pyramidal neurons. At the end of behavioral testing, electrophysiological (neural cellular excitability and Excitatory Post-Synaptic Currents) and morphological (spinogenesis) correlates were evaluated in the PrL pyramidal neurons. Furthermore, transcriptomic cell-specific RNA-analyses (differential gene expression profiling and functional enrichment analyses) were performed in amygdala pyramidal neurons. Our results show that the optogenetic activation of PrL pyramidal neurons in fear-conditioned mice induces fear extinction deficits, reflected in an increase of cellular excitability, excitatory neurotransmission, and spinogenesis of PrL pyramidal neurons, and associated to strong modifications of the transcriptome of amygdala pyramidal neurons. Understanding the electrophysiological, morphological, and transcriptomic architecture of fear extinction may facilitate the comprehension of fear-related disorders.

## 1. Introduction

In variable and challenging environments with various contextual situations, the individuals face a number of approaching dangers. The knowledge of potential threats allows developing fear of threatening situations, choosing among the various behaviors the safest ones, and detecting future dangers. To develop adaptive fear responses, the brain has to discriminate different sensory cues and associate relevant stimuli with aversive events [1]. Thus, when a relevant stimulus (or a context) is associated with an aversive event, fear associations are learned and form a memory.

Learned fear has been widely studied using Contextual Fear Conditioning (CFC), a very useful paradigm to analyze the neuronal and molecular bases of fear associative learning and memory [2,3]. In experimental models in which the CFC paradigm is implemented, the conditioned stimulus (CS), such as a specific cue or context, is associated with the unconditioned stimulus (US), such as foot-shock [3,4]. After the association has taken place, CS alone is able to induce the conditioned response (CR) of fear, such as freezing behavior. The repeated exposure to unreinforced (presented without US) CS gradually weakens the fear CR. In fact, when in the same context the danger is no more present, fear memory goes extinct and other survival functions may be implemented. Interestingly, extinguishing a fear response does not simply involve the fading away of the previous learning. Rather, during extinction process the subject learns something new - the cue no longer predicts that the fearful event will occur [5]. Notably, the impairment of the coping mechanisms dampening fear memory results in maladaptive behaviors. Compromised fear extinction is the key clinical feature of several fear-related disorders, such as post-traumatic stress disorders (PTSD), generalized anxiety, major depression, and phobias [5]. The acquisition of CS-US associative memory as well as the acquisition and maintenance of extinction memory require coordinated neural activity within the fear matrix, encompassing amygdala, medial prefrontal cortex (mPFC), and hippocampus [6,7,8,9,10,11]. Specifically, the activation of pyramidal neurons of the basolateral amygdala (BLA) is necessary to associate sensory input with US [12,13]. Interacting with the amygdala, the mPFC exerts top-down control allowing for appropriate fear responses. In this framework, it has been demonstrated that the balance between expression and extinction of fear CR is modulated by bidirectional inputs from/to the amygdala to/from two sub-regions of the mPFC: the prelimbic (PrL) cortex, which promotes fear responses, and the infralimbic (IL) cortex, which promotes fear extinction [14,15]. In literature are reported in vivo optogenetic manipulations performed by stimulating or inhibiting, with advanced spatial and temporal precision, specific neurons in the fear matrix. These studies demonstrate that the balanced firing activity between amygdala and mPFC is causally involved in fear processing [14,16,17,18,19,20]. Fear extinction decreases the efficacy of excitatory synaptic transmission in the projections from mPFC to amygdala, and promotes the inhibition in mPFC-amygdala pathway [21]. In parallel, projections from the amygdala to PrL cortex exhibit cell-type-specific plasticity during states of high fear, whereas projections from the amygdala to IL cortex are activated during states of low fear [19].

It has to be noted that any behavior has a specific genomic, transcriptomic, and epigenetic signature. Namely, transcriptomic changes are a crucial component of the neuronal modifications that underlie learning and memory [22,23,24], also after the fear exposure [25,26,27]. To date, specific genes involved in functional plasticity and synaptic wiring during fear memory are retained possible disease contributors and potential therapeutic targets for fear-related disorders [28,29,30,31,32]. The environmental experiences may modify the transcriptome [33,34,35,36], and these modifications, in turn, may explain the variability in resilience or predisposition to fear-related disorders as well as the severity of their symptomatology [37]. Understanding the transcriptomic architecture of compromised fear inhibition may thus facilitate the comprehension of fear-related disorders and the identification of potential therapeutic targets.

In the present study, adult transgenic mice, endowed with light-activated ion channel-expressing pyramidal neurons, were used. These transgenic mice were submitted to CFC receiving (or not) the US, to test the synergy between the activity of the amygdala and mPFC in fear learning. In vivo optogenetic manipulations (photo-activations) of the PrL pyramidal neurons were delivered during fear extinction. At the end of behavioral testing, electrophysiological, morphological, and transcriptomic correlates were evaluated. Namely, in PrL pyramidal neurons neural cellular excitability, excitatory neurotransmission, and spinogenesis were evaluated. In parallel, amygdala pyramidal neurons were sorted to perform cell-specific RNA-analyses (differential gene expression profiling and functional enrichment analysis). This methodology permitted conducting a genome-wide investigation of pyramidal neuron expression patterns without a priori selection of specific gene factors, and preventing bias in gene expression brought by bulk tissue analysis and biological subject pooling. Notably, investigating the gene expression of amygdala pyramidal neurons revealed the specific transcriptional pathways associated to impaired fear extinction.

## 2. Results

### 2.1. Behavioral Results: In Vivo Optogenetics of the PrL Pyramidal Neurons during CFC

**Figure 1:**
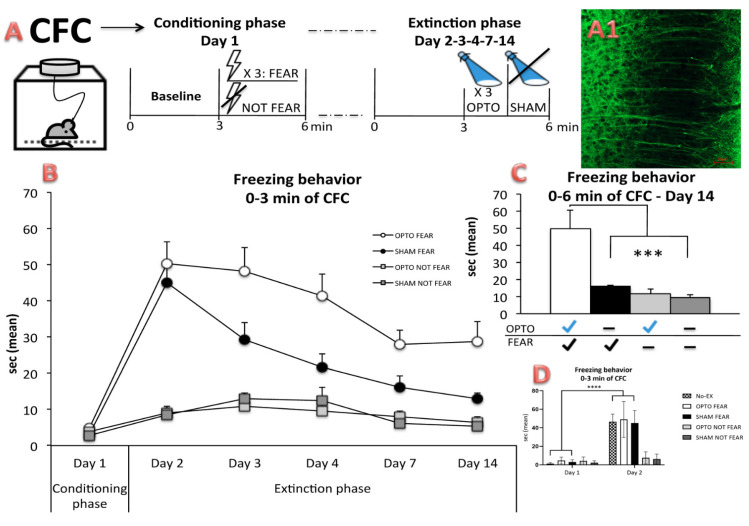
Experimental procedures and behavioral results of in vivo optogenetics of the Prelimbic (PrL) pyramidal neurons during Contextual Fear Conditioning (CFC). (**A**) On day 1 (Conditioning phase) of CFC, each Thy1-COP4 mouse was allowed to explore the conditioning chamber for 3 min (Baseline). Afterward, only a part of the entire sample received three foot-shocks. On days 2, 3, 4, 7, and 14 (Extinction phase), the fear-conditioned (*n* = 20) and not fear-conditioned (*n* = 20) mice were placed again in the conditioning chamber for 6 min. During the Extinction phase, no shock was delivered and the mice received three optogenetic (OPTO FEAR and OPTO NOT FEAR groups, *n* = 10/group) or sham (SHAM FEAR and SHAM NOT FEAR groups, *n* = 10/group) stimulations of PrL pyramidal neurons. To control for the effects of learned but not yet extinguished fear, on day 1 (Conditioning phase) of CFC other Thy1-COP4 mice (No-EX group, *n* = 6) were allowed to explore the conditioning chamber for 3 min (Baseline) and then they received three foot-shocks. The day after the Conditioning phase (day 2) these animals were placed again in the conditioning chamber for 6 min (without receiving any optogenetic stimulation) and then sacrificed for electrophysiological and morphological analyses. A1) Representative image of the expression of the transgenic ChR2-YFP fusion protein detected in pyramidal cortical layer 5 neurons of PrL cortex. Scale bar 50 μm. (**B**) Freezing behavior measured during 0–3 min of CFC. The animals belonging to OPTO FEAR, OPTO NOT FEAR, SHAM FEAR, and SHAM NOT FEAR groups showed similar responses in the Conditioning phase and only the fear-conditioned animals (OPTO FEAR and SHAM FEAR groups) showed increased freezing times on day 2. While SHAM FEAR group progressively extinguished fear memories over time, impaired extinction of fear memories was observed in OPTO FEAR group. (**C**) Freezing times measured during 0–6 min of day 14. OPTO FEAR group showed the highest freezing times in comparison to the remaining groups (*** *p* = 0.0005). (**D**) Freezing behavior measured during 0–3 min of day 1 and 2 of CFC. Only the fear-conditioned animals (No-EX, OPTO FEAR, and SHAM FEAR groups) increased their freezing times between day 1 and day 2 (**** *p* < 0.0001), showing similar consolidation of fear memory. Data are reported as mean ± SEM.

The animals were (or not) fear-conditioned by using the CFC with repetitive sessions on day 1 (Conditioning phase), and 2, 3, 4, 7, and 14 (Extinction phase) (Figure 1A). During the extinction phase, the mice received optogenetic (OPTO FEAR and OPTO NOT FEAR groups) or sham (SHAM FEAR and SHAM NOT FEAR groups) stimulations. A three-way ANOVA (stimulation × fear × day) on freezing behavior (measured during 0–3 min of CFC) (Figure 1B, data expressed in percentage of freezing times are showed in Supplementary Figure S1A) revealed significant stimulation (F_1,36_ = 6.13; *p* = 0.018), fear (F_1,36_ = 63.29; *p* < 0.0001), and day (F_5,36_ = 50.27; *p* < 0.0001) effects. The first-order interactions (at least *p* = 0.05), as well as the second-order interaction (stimulation × fear × day) (F_5,180_ = 3.00; *p* = 0.013) were significant. As revealed by Newman-Keuls post-hoc comparisons, while all animals showed similar responses in the 0–3 min of Conditioning phase, evidence of consolidation was seen on the first extinction day only in the fear-conditioned animals (OPTO FEAR and SHAM FEAR groups), as revealed by their significantly increased freezing times between the Conditioning phase and day 2 (*p* = 0.00004 for both OPTO FEAR and SHAM FEAR groups), and by the similar freezing times of the two groups (*p* = 0.90). As expected, SHAM FEAR animals progressively extinguished fear memories over time and on day 14 their freezing behavior returned to a level similar (*p* = 0.17) to that showed during the Conditioning phase. Interestingly, OPTO FEAR group showed impaired extinction of fear memories. On day 14, OPTO FEAR mice still displayed freezing times significantly longer than those of the Conditioning phase (*p* = 0.00002). One-way ANOVA on freezing behavior (measured during 0–6 min of day 14) of all groups revealed a significant group effect (F_3,36_ = 11.05; *p* = 0.00003) (Figure 1C, data expressed in percentage of freezing times are showed in Supplementary Figure S1B). As revealed by Newman-Keuls post-hoc comparisons, OPTO FEAR group showed the highest freezing times in comparison to the remaining groups (at least *p* = 0.0005).

To control for the effects of learned but not yet extinguished fear, other mice (No-EX group) were fear-conditioned by using the CFC paradigm without the extinction protocol. Freezing times in the 0–3 min of Conditioning phase and day 2 shown by No-EX group were similar to those shown by OPTO FEAR and SHAM FEAR groups, in the same time-points. A two-way ANOVA (group × day) on freezing times (measured during 0–3 min of CFC) revealed significant group (F_4,41_ = 24.57; *p* < 0.0001) and day (F_1,41_ = 268.50; *p* < 0.00001) effects. The interaction was significant (F_4,41_ = 35.87; *p* < 0.0001). As revealed by Newman-Keuls post-hoc comparisons, only the fear-conditioned animals (No-EX, OPTO FEAR, and SHAM FEAR groups) increased their freezing times between day 1 and day 2, showing similar consolidation of fear memory.

### 2.2. Electrophysiological Results: Cellular Excitability of PrL Pyramidal Neurons

To test the ability of the optogenetics to modulate the activity of PrL cortex in fear-conditioned or not fear-conditioned animals, an extensive characterization of the cellular excitability of pyramidal layer 5 neurons after sham or optogenetic stimulation was performed.

**Figure 2:**
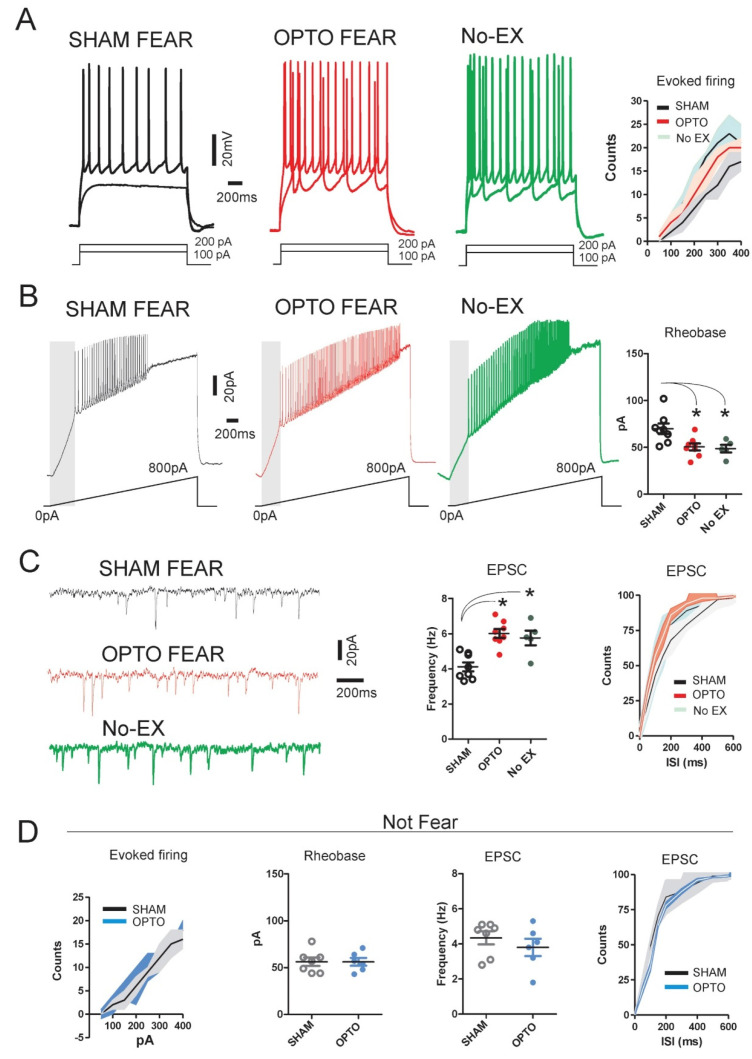
Modulation of cellular excitability of Prelimbic (PrL) pyramidal neurons by optogenetic stimulation. (**A**) Representative traces in current-clamp configuration reporting evoked firing activity triggered by a series of depolarizing current steps (0 to 400 pA) applied to PrL pyramidal neurons of SHAM FEAR (black, *n* = 8 neurons from 5 mice), OPTO FEAR (red, *n* = 8 neurons from 5 mice), and No-EX (green, *n* = 5 neurons from 3 mice) groups. The cumulative plot shows the changes in firing activity. (**B**) Representative traces of PrL pyramidal neurons of SHAM FEAR (black, *n* = 8 neurons from 5 mice), OPTO FEAR (red, *n* = 8 neurons from 5 mice), and No-EX (green, *n* = 5 neurons from 3 mice) groups showing the firing activity triggered by linear depolarization from 0 to 800 pA. Graph (on the right) reports the effects of optogenetic stimulation on rheobase value. Namely, PrL pyramidal neurons of OPTO FEAR and No-EX groups recorded after optogenetic stimulation showed a clear reduction in the rheobase value in comparison to neurons of SHAM FEAR group (* at least *p* = 0.01). (**C**) Representative traces of Excitatory Post-Synaptic Currents (EPSC) of PrL pyramidal neurons of SHAM FEAR (black, *n* = 8 neurons from 5 mice), OPTO FEAR (red, *n* = 8 neurons from 5 mice), and No-EX (green, *n* = 5 neurons from 3 mice) groups. Graph plot (in the middle) and cumulative curve (on the right) depict the clear increase in firing frequency in PrL pyramidal neurons of OPTO FEAR and No-EX groups (* at least *p* = 0.01). (**D**) Graphs and cumulative curves report no significant differences in cellular excitability in PrL pyramidal neurons of SHAM NOT FEAR (black) and OPTO NOT FEAR (blue) groups. Data are reported as median with interquartile range.

In OPTO FEAR group, the optogenetic stimulation induced a robust increase in the evoked firing activities triggered by growing pulses of depolarizing current (0 to 400 pA). Neurons of OPTO FEAR animals clearly showed a higher number of action potentials in comparison to neurons of SHAM FEAR animals, at all current levels considered as indicated by cumulative curve (Correlation test, SHAM FEAR group: Pearson *r =* 0.99, *R*^2^ = 0.99, *n* = 8 neurons from 5 mice; OPTO FEAR group: Pearson *r =* 0.98, *R*^2^ = 0.97, *n* = 8 neurons from 5 mice; *p* < 0.0001, Figure 2A). Rheobase that represents the lowest current amplitude able to generate an action potential is a further useful parameter to investigate changes in neural excitability. PrL pyramidal neurons of OPTO FEAR group recorded after optogenetic stimulation showed a clear reduction in the rheobase value in comparison to neurons of SHAM FEAR group (SHAM FEAR group: 69.88 ± 5.5 pA, *n* = 8 neurons from 5 mice, OPTO FEAR group: 50.50 ± 3.7 pA, *n* = 8 neurons from 5 mice, Mann-Whitney U Test *p* = 0.002; Figure 2B). To test the effect of optogenetic stimulation upon glutamatergic input to pyramidal neurons, the Excitatory Post-Synaptic Currents (EPSC) have been recorded in OPTO FEAR and SHAM FEAR groups. To avoid any inhibitory current contamination, the recordings have been made under pharmacological isolation by bath application of the GABAA blocker, Picrotoxin (10 min, 50 μM). Pyramidal PrL neurons of OPTO FEAR group showed a significantly higher frequency with respect to neurons of SHAM FEAR group (SHAM FEAR group: 4.11 ± 0.26 Hz, *n* = 8 neurons from 5 mice, OPTO FEAR group: 6.01 ± 0.25 Hz, *n* = 8 neurons from 5 mice, Mann-Whitney U Test *p* = 0.002, Figure 2C). Cumulative plot clearly corroborated such an effect (Correlation test, SHAM FEAR group: Pearson *r =* 0.85, *R*^2^ = 0.72, *n* = 8 neurons from 5 mice; OPTO FEAR group: Pearson *r =* 0.75, *R*^2^ = 0.56, *n* = 8 neurons from 5 mice, *p* = 0.0004, Figure 2C). No significant differences in EPSC amplitudes were found between SHAM FEAR and OPTO FEAR groups (SHAM FEAR group: 17.24 ± 0.81 pA, *n* = 8 neurons from 3 mice; OPTO FEAR group: 18.31 ± 0.69 pA, *n* = 8 neurons from 4 mice, Mann-Whitney U Test *p* = 0.37, data not shown).

To control for the effects of learned but not yet extinguished fear on cellular excitability, electrophysiological data recorded from PrL pyramidal neurons of No-EX group were compared with those of OPTO FEAR and SHAM FEAR groups. The excitability of PrL neurons in No-EX mice appeared significantly higher when compared with that showed by SHAM FEAR mice and quite similar to that showed by OPTO FEAR mice. In particular when compared with SHAM FEAR mice, No-EX mice showed an increased evoked firing activities (No-EX group Correlation test: Pearson *r =* 0.96, *R*^2^ = 0.92, *n* = 6 neurons from 3 mice; *p* < 0.0001, Figure 2A), a lower rheobase value (No-EX group: 48.60 ± 4.1 pA, *n* = 5 neurons from 3 mice; Mann-Whitney U Test *p* = 0.01; Figure 2B) and an higher EPSC frequency (No-EX group: 5.77 ± 0.42 Hz, *n* = 5 neurons from 3 mice, Mann-Whitney U Test *p* = 0.01, Figure 2C).

Conversely, in both groups of not fear-conditioned animals (SHAM NOT FEAR and OPTO NOT FEAR groups) the optogenetic stimulation did not produce any significant difference in cellular excitability. Neither the evoked firing nor the rheobase nor EPSC frequency showed any significant difference between SHAM NOT FEAR and OPTO NOT FEAR groups (Figure 2D).

Taken together these data demonstrate that optogenetic stimulation was able to modify the intrinsic cellular excitability of PrL pyramidal neurons supporting the assumption that this area is involved in modulation of the fear responses during extinction phase.

### 2.3. Morphological Results: Spine Counting of PrL Pyramidal Neurons

**Figure 3:**
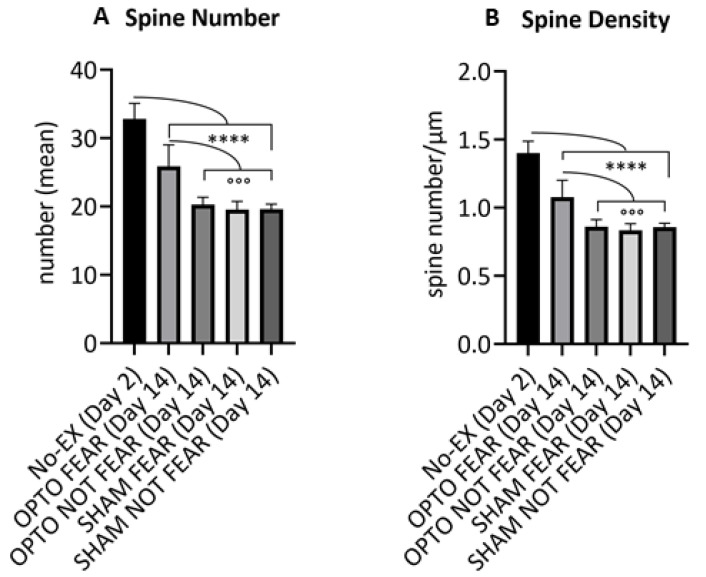
Spine counting of the apical arborizations of Prelimbic pyramidal neurons. No-EX group, encompassing animals submitted to fear learning but not to fear extinction (sacrificed at Day 2), had the highest number (**A**) and density (**B**) of dendritic spines in comparison to OPTO FEAR, OPTO NOT FEAR, SHAM FEAR, and SHAM NOT FEAR groups (**** at least *p* = 0.0001). The OPTO FEAR group showed higher spine number (**A**) and density (**B**) in comparison to the other groups (°°° at least *p* = 0.0001) that, in turn, exhibited similar spinogenesis. Data are reported as mean ± SEM.

The five experimental groups exhibited different spine number and density in apical arborizations of PrL pyramidal neurons (Figure 3), as demonstrated by one-way ANOVAs on number (F_4,18_ = 34.47; *p* < 0.0001) and density (F_4,18_ = 37.15; *p* < 0.0001) of dendritic spines. Newman-Keuls post-hoc comparisons indicated that No-EX group had the highest number and density of dendritic spines in comparison to the other groups (at least *p* = 0.00001). Furthermore, OPTO FEAR group showed higher spine number and density in comparison to the other groups (at least *p* = 0.0001) that, in turn, exhibited similar spine number and density.

### 2.4. Transcriptomic Results: Differential Gene Expression Profiling and Functional Enrichment Analysis of RNA Extracted by Sorted Amygdala Pyramidal Neurons

**Figure 4:**
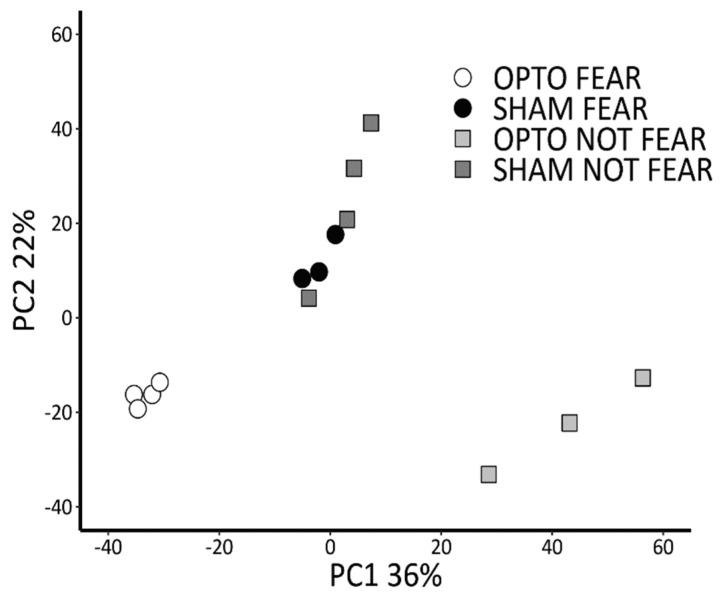
Principal Component Analysis revealing sample clustering based on gene expression profiles. While gene expression profile of mice belonging to SHAM FEAR and SHAM NOT FEAR groups appeared clustered, those of individuals belonging to OPTO FEAR and OPTO NOT FEAR groups were markedly segregated.

Gene expression profiling resulted in 4550 protein-coding genes with a reliable expression and they underwent the downstream analysis. A Principal Component Analysis (PCA) was performed to assess sample clustering based on gene expression profiles (Figure 4). Notably, individual samples belonging to the same group clustered well together. Further, while gene expression profiles of mice belonging to SHAM FEAR and SHAM NOT FEAR groups appeared clustered, those of individuals belonging to OPTO FEAR and OPTO NOT FEAR groups were markedly segregated. Differential expression analysis was performed on 2 × 2 design (Group × Condition) and Differentially Expressed Genes (DEGs) were identified. Significant differentially expressed genes were identified for a *q* > 0.95, equivalent to an FDR-corrected *p* < 0.05. Subsequently, Gene Ontology (GO) and Kyoto Encyclopedia of Genes and Genomes (KEGG) annotations and over-representation analyses (ORA) were performed using clusterProfiler (extended results are reported as Supplementary Results 1 and in Supplementary Figure S2). Significant pathways were shown by means of enrichment map method.

#### 2.4.1. Comparison between OPTO FEAR vs. SHAM FEAR Groups

**Figure 5:**
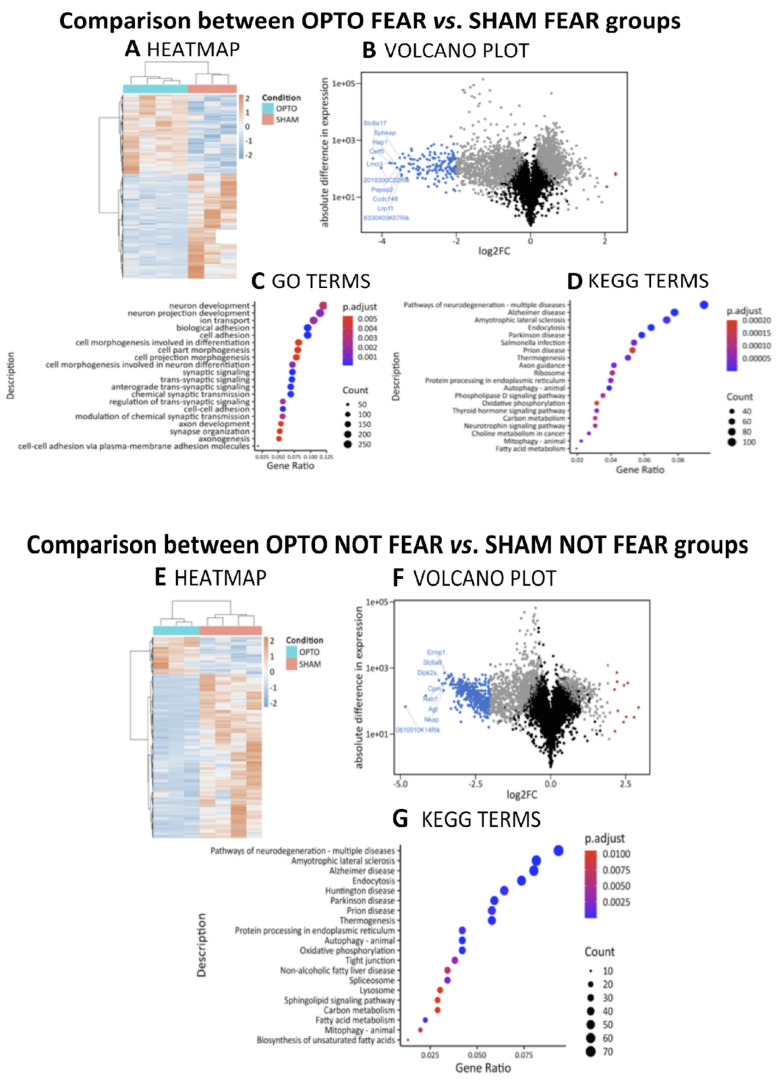
Differential Gene Expression profiling and functional enrichment analysis of RNA extracted by amygdala pyramidal neurons. Comparisons between OPTO FEAR vs. SHAM FEAR groups (upper part) and OPTO NOT FEAR vs. SHAM NOT FEAR groups (lower part). (**A**,**E**) Heatmaps showing gene expression values for the Differentially Expressed Genes (DEGs). (**B**,**F**) Volcano plots highlighting DEGs. The *x*-axis is the log2 fold change (log2FC) in normalized gene expression and the *y*-axis is for the log_10_ absolute value of the difference in expression between conditions. Each dot represents a gene. Grey dots are for DEGs, blue and red dots are for <−2 and >+2 log2FC genes, respectively. The top ten genes with the highest absolute log2FC values are labeled. (**C**,**D**,**G**) Dot plots representing the top twenty enriched terms from over-representation analyses (ORA) in Gene Ontology (GO) and Kyoto Encyclopedia of Genes and Genomes (KEGG) databases. GO terms from different domains (Biological Processes, Cellular Component, and Molecular Function) were sorted by *q*-value before plotting them together.

Differential expression analysis showed 2417 significant DEGs (1043 up; 1374 down, with reference level set on the SHAM FEAR condition) (Figure 5A,B, Table 1). To explore further the biological significance of the transcriptomic modulations caused by optogenetic stimulation in the presence of fear memory, ORA was performed on the obtained DEGs and resulted in 137 significantly enriched GO (Biological Processes, BP: 57; Cellular Component, CC: 61; Molecular Function, MF: 19) terms and 132 significantly enriched KEGG terms. The top twenty significant GO terms belonged to BP and CC and highlighted a differential involvement of the pathways associated with neuronal plasticity and synaptic signaling, resulting in an overall modulation of synaptic organization (Figure 5C) (namely: GO:0098590 Plasma membrane region, GO:0031226 Intrinsic component of plasma membrane, GO:009897 Glutamatergic synapse, GO:0005887 Integral component of plasma membrane, GO:0044456 Synapse part, GO:0030424 Axon, GO:0042734 Presynaptic membrane, GO:0031253 Cell projection membrane, GO:0098794 Postsynapse, GO:0099537 Trans-synaptic signaling, GO:0098609 Cell–cell adhesion, GO:0007155 Cell adhesion, GO:0099536 Synaptic signaling, GO:0022610 Biological adhesion, GO:0007268 Chemical synaptic transmission, GO:0098916 Anterograde trans-synaptic signaling, GO:0030425 Dendrite, GO:0097447 Dendritic tree, GO:0099572 Postsynaptic specialization, and GO:0097060 Synaptic membrane).

The top twenty significant KEGG terms resulted associated to signaling processes related to several neurodegenerative diseases and metabolic pathways (Figure 5D) (namely: mmu05022 Pathways of neurodegeneration—multiple diseases, mmu04144 Endocytosis, mmu05010 Alzheimer Disease, mmu05012 Parkinson Disease, mmu04140 Autophagy—animal, mmu05014 Amyotrophic Lateral Sclerosis, mmu04137 Mitophagy—animal, mmu05132 Salmonella infection, mmu04714 Thermogenesis, mmu04360 Axon guidance, mmu04919 Thyroid hormone signaling pathway, mmu04141 Protein processing in endoplasmic reticulum, mmu05231 Choline metabolism in cancer, mmu04722 Neurotrophin signaling pathway, mmu03010 Ribosome, mmu04072 Phospholipase D signaling pathway, mmu01212 Fatty acid metabolism, mmu01200 Carbon metabolism, mmu05020 Prion disease, and mmu00190 Oxidative phosphorylation).

#### 2.4.2. Comparison between OPTO NOT FEAR vs. SHAM NOT FEAR Groups

Differential expression analysis showed 1661 significant DEGs (308 up; 1353 down, with reference level set on the SHAM NOT FEAR condition) (Figure 5E,F; Table 2). The ORA performed on the obtained DEGs resulted only in 36 significantly enriched KEGG terms and 0 significantly enriched GO terms. Here, the genes universe did not allow enriching many GO terms because of sampling bias correction [38]. Again, the top twenty over-represented KEGG terms were related to neurodegenerative diseases and metabolic pathways (Figure 5G) (namely: mmu04144 Endocytosis, mmu00190 Oxidative phosphorylation, mmu04140 Autophagy—animal, mmu05014 Amyotrophic Lateral Sclerosis, mmu04714 Thermogenesis, mmu05010 Alzheimer Disease, mmu05012 Parkinson Disease, mmu05022 Pathways of neurodegeneration—multiple diseases, mmu05016 Huntington Disease, mmu01212 Fatty acid metabolism, mmu05020 Prion Disease, mmu04141 Protein processing in endoplasmic reticulum, mmu03040 Spliceosome, mmu04530 Tight junction, mmu04137 Mitophagy—animal, mmu01040 Biosynthesis of unsaturated fatty acids, mmu04932 Non-alcoholic fatty liver disease, mmu04142 Lysosome, mmu01200 Carbon metabolism, and mmu04071 Sphingolipid signaling pathway).

#### 2.4.3. Comparison between OPTO FEAR vs. OPTO NOT FEAR Groups

**Figure 6:**
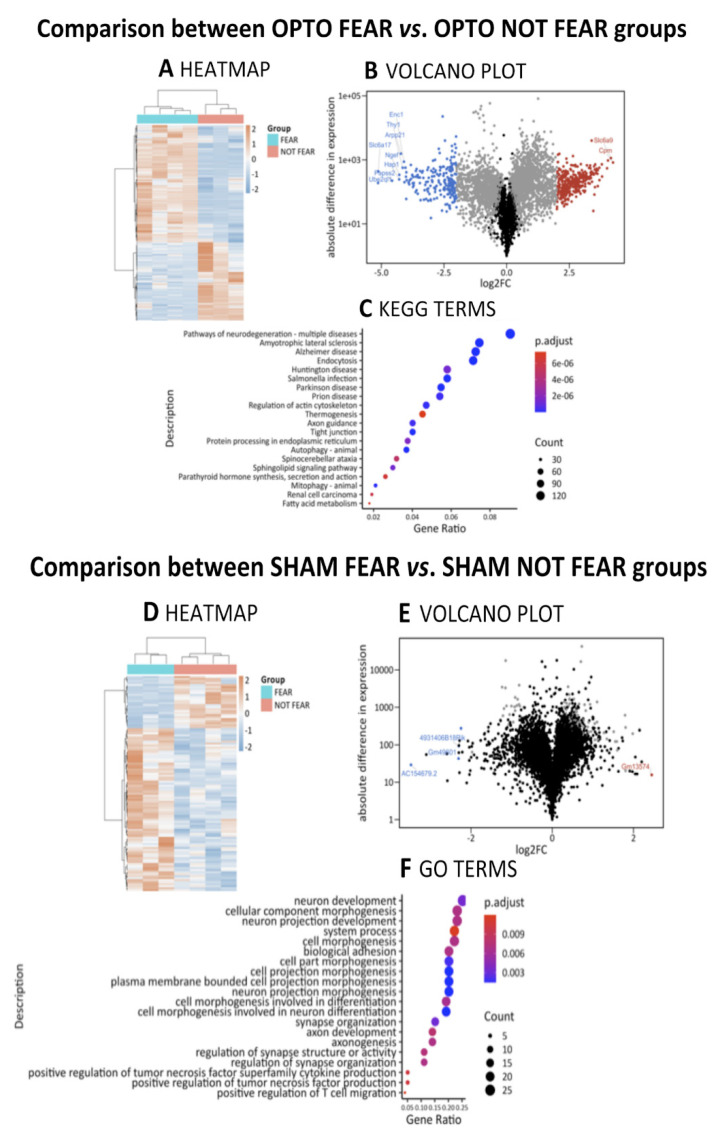
Differential Gene Expression profiling and functional enrichment analysis of RNA extracted by amygdala pyramidal neurons. Comparisons between OPTO FEAR vs. OPTO NOT FEAR groups (upper part) and SHAM FEAR vs. SHAM NOT FEAR groups (lower part). (**A**,**D**) Heatmaps showing gene expression values for the Differentially Expressed Genes (DEGs). (**B**,**E**) Volcano plots highlighting DEGs. The *x*-axis is the log2 fold change (log2FC) in normalized gene expression and the *y*-axis is for the log_10_ absolute value of the difference in expression between conditions. Each dot represents a gene. Grey dots are for DEGs, blue and red dots are for <−2 and > + 2 log2FC genes, respectively. The top ten genes with the highest absolute log2FC values are labeled. (**C**,**F**) Dot plots representing the top twenty enriched terms from over-representation analyses (ORA) in Gene Ontology (GO) and Kyoto Encyclopedia of Genes and Genomes (KEGG) databases. GO terms from different domains (Biological Processes, Cellular Component, and Molecular Function) were sorted by *q*-value before plotting them together.

Differential expression analysis showed 3506 significant DEGs (2104 up; 1402 down, with reference level set on the OPTO NOT FEAR condition) (Figure 6A–C, Table 3). Despite the high number of DEGs, the ORA performed on the obtained DEGs resulted only in 3 significantly enriched GO terms (BP: 0; MF: 0; CC: 3, i.e.,: GO:0005887 integral component of plasma membrane, GO:0031226 intrinsic component of plasma membrane, and GO:0098590 plasma membrane region), indicating a broad modulation of genes without a significant enrichment for specific pathways. These large differences in transcriptome include crucial pathways linked to neuron morphogenesis and differentiation (e.g., GO:0031175 Neuron projection development, GO:0048667 Cell morphogenesis involved in neuron differentiation, GO:0007409 Axonogenesis, GO:0061564 Axon development, GO:0000904 Cell morphogenesis involved in differentiation) (Supplementary Results 1).

In parallel, 169 KEGG terms resulted significantly enriched. The top twenty over-represented KEGG terms were associated to neurodegeneration, metabolic regulation, regulation of actin cytoskeleton, and cancer (namely: mmu04144 Endocytosis, mmu05132 Salmonella infection, mmu05022 Pathways of neurodegeneration—multiple diseases, mmu04140 Autophagy—animal, mmu05014 Amyotrophic Lateral Sclerosis, mmu05012 Parkinson Disease, mmu05010 Alzheimer Disease, mmu04530 Tight junction, mmu04137 Mitophagy—animal, mmu04810 Regulation of actin cytoskeleton, mmu05020 Prion disease, mmu04360 Axon guidance, mmu04071 Sphingolipid signaling pathway, mmu05016 Huntington Disease, mmu04141 Protein processing in endoplasmic reticulum, mmu05017 Spinocerebellar ataxia, mmu05211 Renal cell carcinoma, mmu04928 Parathyroid hormone synthesis, secretion and action, mmu01212 Fatty acid metabolism, and mmu04714 Thermogenesis).

#### 2.4.4. Comparison between SHAM FEAR vs. SHAM NOT FEAR Groups

In line with PCA highlighting poor segregation between SHAM FEAR vs. SHAM NOT FEAR groups, differential Expression analysis showed only 107 significant DEGs (81 up; 26 down, with reference level set on SHAM NOT FEAR condition) (Figure 6D,E, Table 4). Despite the small number of DEGs, the ORA performed on the obtained DEGs resulted in 129 enriched GO terms (BP: 67; CC: 45; MF: 17) and 3 enriched KEGG terms. The top twenty significant GO terms belonged to BP and CC and suggested the differential involvement of pathways related to synaptic plasticity, in particular related to excitatory synapse, and morphogenesis (Figure 6F) (namely: GO:0009986 Cell surface, GO:0048471 Perinuclear region of cytoplasm, GO:0030424 Axon, GO:0036477 Somato-dendritic compartment, GO:0030425 Dendrite, GO:0097447 Dendritic tree, GO:0043025 Neuronal cell body, GO:0098590 Plasma membrane region, GO:0048812 Neuron projection morphogenesis, GO:0048667 Cell morphogenesis involved in neuron differentiation, GO:0120039 Plasma membrane bounded cell projection morphogenesis, GO:0048858 Cell projection morphogenesis, GO:0032279 Asymmetric synapse, GO:0032990 Cell part morphogenesis, GO:0060076 Excitatory synapse, GO:0005887 Integral component of plasma membrane, GO:0098984 Neuron to neuron synapse, GO:0044456 Synapse part, GO:0097038 Perinuclear endoplasmic reticulum, and GO:0033267 Axon part). KEGG analysis showed modulation of the pathways associated to cAMP signaling (mmu04024), Prostate cancer (mmu05215), and Thyroid hormone signaling (mmu04919).

#### 2.4.5. DEGs Involved in Learning/Memory and Fear Response in the Comparison between OPTO FEAR vs. SHAM FEAR Groups

Despite the relative pathways were not identified as significantly enriched, we wondered whether genes involved in learning/memory and fear response were significantly modulated in the case of impaired fear extinction caused by the optogenetic stimulation on PrL pyramidal neurons.

**Figure 7:**
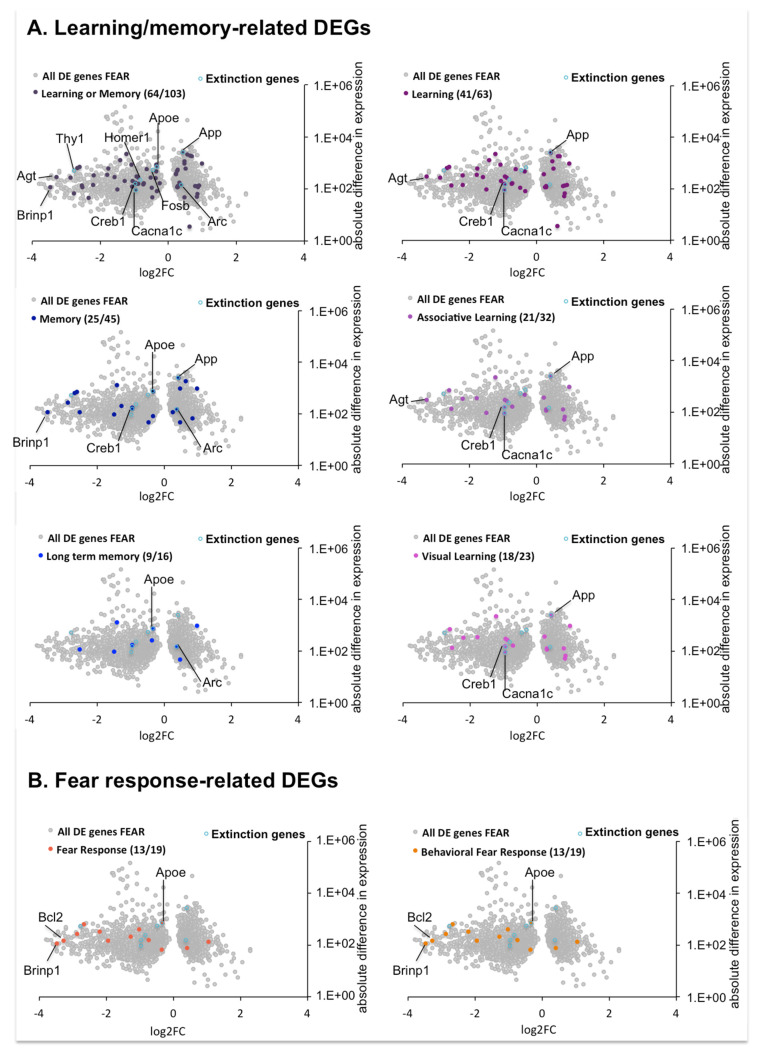
Differentially Expressed Genes (DEGs) with Gene Ontology annotations associated to Learning/memory and Fear response, between OPTO FEAR vs. SHAM FEAR groups. DEGs for each indicated Biological Process (BP) are highlighted as colored dots on the volcano plot representing the total DEGs between OPTO FEAR vs. SHAM FEAR groups, indicated by grey dots. Numbers into brackets indicate the number of DEGs related to each indicated BP over the gene universe. Light blue dots indicate genes described in literature as associated to fear extinction. When DEGs are part of the top twenty DEGs list and/or part of the considered BP the gene name is indicated.

By looking at genes with GO annotations related to BP associated to Learning/memory and Fear response, we identified several DEGs between OPTO FEAR vs. SHAM FEAR groups (with reference level set on the SHAM FEAR condition; genes related to Learning/memory: Learning or Memory, 64 over 103 from the gene universe, Learning, 41 over 63, Memory, 25 over 45, Associative Learning, 18 over 23, Long-term memory, 9 over 16, and Visual Learning, 18 over 23; genes related to Fear response: Fear Response and Behavioral Fear Response, 13 over 19) (Figure 7, Supplementary Results 2). This set contains 3 of the top twenty DEGs, all down regulated (with the log2 Fold Change (FC) ranging from −4.24 to −3.23): *Brinp1* (associated to Fear response and Memory), *Bcl2* (associated to Fear response), and *Agt* (associated to Learning) in the comparison between OPTO FEAR and SHAM FEAR groups. We also identified several genes well known to be associated with fear extinction: *ApoE*, *Cacna1* (*Cav1.2*), *Creb1*, *App*, and *Arc*. Most of them appeared to be down regulated in the comparison between OPTO FEAR vs. SHAM FEAR groups, but to a lesser extent (*Apoe* log2FC = −0.33; *Cacna1c* (*Cav1.2*) log2FC = −0.94; *Creb1* log2FC = −0.96), while we noted a mild but significant increase in the expression of *App* (log2FC = +0.41) and *Arc* (log2FC = 0.37) genes. Despite non-being a gene with GO annotations related to BP associated to learning/memory and fear response, we noted that *Homer1*, another gene recently associated to fear memory extinction, appeared significantly down regulated (log2FC = −0.87), in the comparison between OPTO FEAR vs. SHAM FEAR groups. Finally, we also noted the strong down regulation of *Thy1* (log2FC = −2.77) gene.

## 3. Discussion

Starting from the assumption that fear learning and extinction are adaptive processes caused by molecular changes in the amygdala-mPFC neural circuit, in the present research adult mice were submitted to CFC receiving (or not) the aversive US as well as the optogenetic (or sham) stimulations to maintain the activation of the PrL pyramidal neurons and impair fear extinction. At the end of behavioral testing, cellular excitability and excitatory neurotransmission, as well as spinogenesis, were evaluated in PrL pyramidal neurons. In parallel, amygdala pyramidal neurons were sorted to perform cell-type specific RNA sequencing, able to reveal the specific transcriptional signature associated to impaired fear extinction.

All fear-conditioned animals (OPTO FEAR, SHAM FEAR, and No-EX groups) showed consolidation of aversive memory. However, while SHAM FEAR group progressively extinguished fear memory as expected, impaired fear extinction was observed in OPTO FEAR group (Figure 1 and Supplementary Figure S1).

Once reactivated through the re-exposure to the context, the memory of fear conditioning becomes susceptible to be modified [39]. In fact, the re-exposure to the context elicits a series of processes (retrieval, reconsolidation, and extinction), which are governed by “boundary conditions” [39,40]. While extinction refers to the decrement of fear CR that occurs with repeated presentations of CS (no more reinforced with US), and reflects the formation of a new inhibitory memory, reconsolidation is a process whereby previously consolidated memories can be reactivated and again made sensitive to mutate. Notably, the manipulations during or shortly after the period of memory reactivation can influence reconsolidation process. Thus, the present optogenetic stimulations of the PrL pyramidal neurons applied from 3rd min onwards of each day of Extinction phase allowed reducing extinction learning. Although the reconsolidation and extinction are distinct (even if interrelated) processes, distinguishing the role of PrL in the transition between them was out of the scope of the present work.

Interestingly, in fear-conditioned mice the optogenetic stimulation induced a robust increase in evoked firing activity, number of action potentials, EPSC frequency (Figure 2), as well as number and density of dendritic spines in the apical arborizations of PrL pyramidal neurons (Figure 3). Conversely, in not fear-conditioned mice the optogenetic stimulation did not produce any significant change in cellular excitability, evoked firing, EPSC frequency, and spinogenesis (Figure 2 and Figure 3). To better control the effects of learned but not yet extinguished fear, electrophysiological and morphological data of PrL pyramidal neurons from No-EX group were compared with those of the other groups. The findings indicate that No-EX group that had consolidated fear memory, exhibited an excitability of PrL neurons higher than that shown by PrL neurons of SHAM FEAR mice and similar to the excitability shown by OPTO FEAR mice. Furthermore, No-EX mice displayed highest number and density of dendritic spines in apical arborizations of PrL pyramidal neurons. The results demonstrated that while the fear conditioning increased the excitability and morphological properties of PrL pyramidal neurons, the fear extinction decreased them. Optogenetic activation of PrL pyramidal neurons counteracted such reductions, thus leading to impaired extinction.

It has been proposed that the boost of bidirectional amygdala-PrL synaptic transmission leads to changes in the representation of learned CS-US association in mPFC neurons [19,20]. Optogenetic experiments have confirmed that modified synaptic transmission in the amygdala-PrL network during fear learning interferes with long-term fear consolidation [14]. By using ex vivo electrophysiological recordings, combined with optogenetic techniques, it has been demonstrated that fear extinction decreases the efficacy of excitatory transmission from mPFC to the amygdala [21]. In parallel, the amygdala neurons projecting to mPFC exhibit cell-type-specific plasticity during fear extinction [19].

Cell-type-specific transcriptome analysis represents cutting-edge tool to reveal targets useful for understanding and treating fear-related disorders. Differential gene expression and co-expression network analyses identified diverse networks activated or inhibited by fear learning vs. extinction, and upstream regulator analysis and viral vector-manipulations demonstrated that fear extinction is associated with reduced cAMP/Ca^2+^ responsive element binding (CREB) expression [41].

Our study identifies cell-type amygdala pyramidal neuron-specific gene networks (by means of the RNA-sequencing) disclosing pathways associated to adaptive or maladaptive fear extinction and opening innovative possibilities to understand deeper the underlying mechanisms of fear process and its impairment. Here we discuss some of the genes and pathways that result as more relevant.

Optogenetic stimulation of the PrL pyramidal neurons was associated with strong modifications of the amygdala pyramidal neuron transcriptome (Figure 5A,B, Table 1). Bioinformatic analyses revealed that among the top twenty DEGs (resulted down regulated in OPTO FEAR mice when compared with SHAM FEAR mice), there were *Gabra4* (Gamma-aminobutyric acid—GABA—A receptor, subunit alpha 4) and *Kcnh3* (potassium voltage-gated channel, subfamily H, member 3), which are associated with synaptic transmission. Namely, shunting inhibition via GABAA receptors reduces activation of N-methyl-D-aspartate receptors, and impairs long-term potentiation [42], as well as voltage-gated potassium channels control cellular excitability by regulating a variety of neuronal properties, such as inter-spike membrane potential, action potential waveform, and firing frequency [43]. Within the brain, *Kcnh3* is expressed in the cerebral cortex, amygdala, hippocampus, and striatal regions, with specific expression in pyramidal neurons [44].

Other DEGs that we identified as associated with impaired extinction were linked to inflammation processes, such as *Csmd1* [45] and *Bcl2* [46]. Notably, overexpression of *Bcl2* blocks the apoptotic death of the pro-B-lymphocyte cells and neurons [47,48]. Furthermore, in the same comparison OPTO FEAR vs. SHAM FEAR, the top twenty GO terms associated with impaired extinction highlighted the differential involvement of pathways associated with neuronal plasticity and glutamatergic synaptic signaling, resulting in modulation of overall pre- and post-synaptic organization. In parallel, the top twenty KEGG terms were associated with processes related to several neurodegenerative diseases, metabolic pathways, production of lipids and proteins, and thyroid hormone and neurotrophin signaling. Remarkably, fear-associated enrichments have been related with dendritic and post-synaptic processes, while extinction-associated enrichments have been related with cellular metabolism and proliferation [41].

By looking at genes related to Learning/memory or Fear response processes, several DEGs were scored in the comparison between OPTO FEAR vs. SHAM FEAR groups (Figure 7). Among these, 3 DEGs (i.e., *Brinp1*, associated to Fear response and Memory; *Bcl2*, associated to Fear response; *Agt*, associated to Learning) were part of the most impacted genes (top twenty DEGs), and resulted strongly down regulated in OPTO FEAR group. Several genes previously associated with fear extinction in the literature [41,49,50,51,52,53,54] were present in OPTO FEAR group, as down regulated (*Apoe*, *Cacna1c*, *Creb1*, *Homer1*) or up regulated (*App*, *Arc*). Although not always the afore-mentioned genes have been associated specifically to amygdala activity, those down regulated have been positively associated with extinction, while those up regulated have been positively associated with fear memory retention.

The photo-stimulation of PrL pyramidal neurons in the presence or absence of fear was differently associated with amygdala pyramidal neuron transcriptome (Figure 6A,B; Table 3). Among the top twenty DEGs, some relevant genes resulted down regulated in OPTO FEAR mice when compared with OPTO NOT FEAR mice. Among these genes, there was *Ngef* (Neuronal guanine nucleotide exchange factor, also known as Ephexin1), an ephrin (Eph) receptor-interacting exchange protein that promotes EphA4 binding and leads to cell morphology changes [55] and reduces spine density [56]. Accordingly, the OPTO FEAR mice (in which *Ngef* is down regulated) exhibited increased number and density of dendritic spines in the apical arborizations. Furthermore, the binding of Eph with its receptors constitutes a molecular link between Eph receptors and actin cytoskeleton and modulates pre-synaptic calcium channel activity [57]. To explore the role of another member of Eph family (EphB2) in memory formation and enhancement, Alapin and colleagues [58] used a photo-activatable EphB2 to activate EphB2 forward signaling in pyramidal neurons of the lateral amygdala. Such a photo-activation during fear learning (but not afterwards) enhances the long-term (but not the short-term) fear response. Accordingly, long-term fear memory is impaired in mice lacking EphB2 forward signaling [58].

Among the top twenty DEGs, another gene associated with actin-binding protein was *Enc1* (ectodermal-neural cortex 1) that resulted down regulated in OPTO FEAR mice. Kim and colleagues [59] showed that expression of *Enc1* induces neuronal process formation, whereas antisense treatment inhibits neurite development. Similarly, *Thy-1* (thymus cell antigen 1) involved in cell–cell interactions, *Ptprg* (protein tyrosine phosphatase, receptor type, G) implicated in the control of cellular proliferation, and *Kcnq2* (potassium voltage-gated channel, subfamily Q, member 2) were down regulated in OPTO FEAR mice. It has been demonstrated that Kcnq2-related proteins are localized on pyramidal neurons, suggesting their pre-synaptic role in action potential propagation and neurotransmitter release [60]. Even *Chrna4* (cholinergic receptor, nicotinic, alpha polypeptide 4) linked to the superfamily of ligand-gated ion channels that mediate fast signal transmission, was down regulated in OPTO FEAR mice. Of note, as an ancillary remark on the extinction process, mice optogenetically stimulated on amygdala cholinergic input during the initial fear learning are more resistant to extinction learning than controls, supporting the role of cholinergic modulation of amygdala circuits in learning and retention of fear memories [61].

Deficit in exploratory behavior and cognitive impairment in learning tasks as well as neuronal death are reported in mutant mice for *Arsg* gene (arylsulfatase G) [62]. Consistently, our analysis shows *Arsg* down regulation in OPTO FEAR mice. Despite the high number (3506) of DEGs, while only 3 GO terms (components of plasma membrane) were enriched, while 169 KEGG terms resulted enriched. The top twenty over-represented KEGG terms were associated with neurodegeneration, metabolism, actin cytoskeleton regulation, and parathyroid hormone regulation.

Optogenetic stimulation of the PrL pyramidal neurons per se, without fear experience, was also associated with a strong down regulation of amygdala gene expression (Figure 5E,F, Table 2). Among DEGs (down regulated in OPTO NOT FEAR mice when compared with SHAM NOT FEAR mice), some genes were implicated in cell proliferation, synaptic activation, and long-term potentiation (such as *Nab1* and *Sos1*), others in inflammation processes (such as *Gpx4* and *Nkap*), with roles in transcriptional repression and RNA splicing and processing (*Nkap*) [63]. Furthermore, in the same comparison the gene universe did not allow enriching many GO terms because of sampling bias correction [38]. Again, the top twenty over-represented KEGG terms were related to neurodegenerative diseases, metabolic pathways, and biosynthesis of protein and unsaturated fatty acids.

Finally, we observed that fear conditioning per se (in the presence of an adaptive extinction), without optogenetic stimulation, was not greatly associated with gene expression in amygdala pyramidal neurons (Figure 6D,E, Table 4), in line with PCA highlighting poor segregation between SHAM FEAR vs. SHAM NOT FEAR groups. This result is in line with those reported by McCullough and colleagues [41] who described a weak separation between the transcriptomes of fear- and not fear-conditioned animals. The authors discussed their findings as result of stress-induced translational changes due to the handling of animals and CS exposure. This hypothesis tested in a separate cohort of mice was confirmed, demonstrating that stress-related genes were similarly regulated in both groups. Thus, it is likely that the signature of associative fear learning was obscured by generalized stress-related changes. Furthermore, while SHAM FEAR group learned the association CS-US, it is not possible to exclude that the SHAM NOT FEAR group encoded the CS (never paired with US). Such a coding might influence or even impede the subsequent acquisition of conditioned associations between the CS and US, as occurs in the case of latent inhibition process. Specifically, latent inhibition refers to the reduced ability to learn the relevance of a stimulus that is paired with an aversive (or positive) condition through classic conditioning if there has been a previous exposure to that stimulus in a neutral context [64,65]. However, the limited segregation between SHAM FEAR and SHAM NOT FEAR gene profiles might also be caused by the time-point of the transcriptomic analyses. In fact, gene profiling of the SHAM FEAR group was performed once the animals had extinguished the fear memories, and fear response was over, although the fear engram was likely still stored in the amygdala-hippocampus-mPFC circuit [66].

Actually, among DEGs present in the comparison between SHAM FEAR vs. SHAM NOT FEAR groups, some genes (such as *Slx4ip* and *Kif6*) were up regulated in SHAM FEAR group and are implicated in DNA/RNA regulation, maintenance, and repair [67,68]. Conversely, the *Ino80* gene resulted down regulated in the same comparison. Notably, it has been reported that the deletion of *Ino80* results in defective cellular proliferation and premature entry into cellular senescence, due to activation of the DNA damage response [69]. Furthermore, other genes associated to metabolism (such as the down regulated *Smcr8* and the up regulated *Clstn3*), neurotransmission (such as the up regulated *Hcn2*), and inflammation (such as the up regulated *Sumf1* and *Plaa*) were found differentially expressed in SHAM FEAR group when compared with SHAM NOT FEAR group. Interestingly, also *Adcyap1r1* (adenylate cyclase activating polypeptide 1 receptor 1) resulted up regulated in SHAM FEAR group. The pituitary adenylate cyclase-activating polypeptide is a hormone that stimulates the secretion of growth hormone, adrenocorticotrophic hormone, catecholamines, and insulin, by its interaction with specific receptors. Interestingly, the methylation of *Adcyap1r1* in peripheral blood has been associated with PTSD, and *Adcyap1r1* mRNA is induced by fear learning [70]. Since DNA methylation of regulatory elements usually acts to repress gene transcription, the findings by Ressler and colleagues [70] indicating that methylation of *Adcyap1r1* is associated with impaired fear extinction (as typically occurring in PTSD) together with the present ones indicating that the up regulation of this gene is associated with efficient fear extinction converge in highlighting that the system pituitary adenylate cyclase-activating polypeptide with its receptors might be an important mediator of abnormal fear responses following trauma [70].

In the same comparison (SHAM FEAR vs. SHAM NOT FEAR), the top twenty GO terms were related to morphogenesis and synaptic plasticity, with the important specificity of the excitatory synapse. KEGG analysis showed modulation of the pathways associated to cAMP signaling, cancer, and thyroid hormone signaling.

Overall, our results show that the optogenetic activation of PrL pyramidal neurons in fear-conditioned mice did not allow the disengagement of the fear matrix, and induced fear extinction deficits mirrored by the increase of cellular excitability, excitatory neurotransmission, and spinogenesis of PrL pyramidal neurons, and by strong modifications of the transcriptome of amygdala pyramidal neurons.

The optogenetic manipulation determined a photo-activation of the pyramidal neurons of layer 5 of PrL cortex projecting to distinct cortical (including IL cortex) and subcortical (including amygdala) targets and allowed deepening the direct (PrL cortex—amygdala) or indirect (PrLcortex—IL cortex—amygdala) crosstalk among structures involved in fear extinction. It is certainly true that the optogenetic stimulation of PrL pyramidal neurons modulated the circuit from mPFC to amygdala, but the effects of PrL optogenetic stimulation were markedly displayed in fear-conditioned animals. This latter observation suggests that the amygdala is the first hub to trigger the fear-associative memory and communicate the fear association to the mPFC.

In optogenetically stimulated fear-conditioned mice the difference in gene expression profiles was characterized by down regulation of genes associated with the synaptic transmission, specifically the inhibitory GABAergic signaling, as well as by differential involvement of pathways associated with neuronal plasticity and glutamatergic signaling.

The present findings indicate that impaired extinction is featured by specific changes of transcriptome that validate previous findings [41], provide targets for future translational research into cell type-specific control of fear extinction, and emphasize the key role of pyramidal neurons belonging to fear matrix. This type of comprehensive cell-type specific analysis may produce an important array of targets potentially useful for diagnosis, treatment, and prevention of fear-related disorders.

### Limitations

For sake of clarity, we would like to report some limitations of the present study that will require future and aimed investigations to be dissolved. First of all, the comparison between amygdala pyramidal neuron transcriptomes of naïve, conditioned (only trained without extinction), and optogenetically stimulated fear-conditioned mice would be an interesting deepening of the present study to definitively clarify what is the role of PrL cortex activation during extinction. It is reasonably conceivable that the amygdala gene expression patterns after fear training (without any optogenetic manipulation and before extinction) are similar to those of mice that underwent PrL optogenetic stimulation and thus showed impaired fear extinction. A strong support (even if not the final demonstration) to this interesting hypothesis derives from the evidence that both cellular excitability (EPSC, evoked firing, and rheobase) and morphological (spine number and density) changes are similar in fear-conditioned (only trained without extinction) mice and in PrL optogenetically stimulated mice showing impaired extinction.

Another point to be considered is that in selecting amygdala pyramidal neurons for transcriptome analyses the used methodology, although innovative and fruitful in its results, does not allow distinguishing whether the sorted neurons are directly or indirectly connected with the PrL cortex. Thus, the reported connections between transcriptome changes in amygdala pyramidal neurons and PrL optogenetic stimulation have to be considered associative and not causal.

As final note, we are aware that although the focused laser beam is very well-suited for spatially-localized optogenetic activation of PrL pyramidal neurons, the scarce out-of-focus light could stimulate other photo-activable neurons very close to PrL pyramidal neurons, reducing thus the spatial resolution. However, it is important to notice that the optogenetic stimulation of PrL cortex elicited the typical behavior derived from PrL activation (as the impaired extinction), and no typical behavior derived from IL activation (as the potentiated extinction). Furthermore, it has to be underlined that OPTO NOT FEAR mice showed no change in behavioral, electrophysiological and structural parameters in comparison to SHAM NOT FEAR mice, suggesting that the PrL photo-activation did not elicit per se any unnatural effect.

## 4. Materials and Methods

### 4.1. Subjects

Male adult (2.5 month-old) B6.Cg-Tg(Thy1-COP4/EYFP)18Gfng/J (Thy1-COP4) (Jackson Laboratories, Bar Harbor, ME, USA) mice were used in the present research. These transgenic mice express the light-activated ion channel, Channelrhodopsin-2 (ChR2), fused to Yellow Fluorescent Protein (YFP) under the control of the mouse thymus cell antigen 1 (*Thy1*) promoter. The expression of the transgenic ChR2-YFP fusion protein is detected in pyramidal cortical layer 5 neurons, in CA1 and CA3 pyramidal neurons of the hippocampus, amygdala pyramidal neurons, cerebellar mossy fibers, neurons in the thalamus, midbrain and brainstem, and olfactory bulb mitral cells. Transgene-expressing neurons are morphologically and physiologically comparable to non-mutant neurons. The ChR2 functions as a blue light-driven cation channel that depolarizes the cell and elicits action potentials. Thus, illuminating ChR2-expressing neurons with blue light (~470 nm) leads to rapid and reversible photo-stimulation evoking action potential firing/neural activity.

The animals were group-housed (4 mice/cage) with food (Mucedola, Milan, Italy) and water ad libitum, and kept under a 12-h light/dark cycle with the light on at 07:00 h, controlled temperature (22–23 °C) and constant humidity (60 ± 5%). All experiments took place during the light phase. All efforts were made to minimize animal suffering and to reduce their number, in accordance with the European Directive (Directive 2010/63/EU). The animals assigned to the same experimental group were never siblings.

### 4.2. Experimental Procedure

Thy1-COP4 mice were unilaterally implanted with a guide cannula on PrL sub-region of right mPFC and then were (or not) fear-conditioned by using the CFC paradigm with the extinction protocol. During the extinction phase of CFC, the mice received optogenetic (OPTO FEAR group, *n* = 10; OPTO NOT FEAR group, *n* = 10) or sham (SHAM FEAR group, *n* = 10; SHAM NOT FEAR group, *n* = 10) stimulations. At the end of the behavioral testing, the animals were sacrificed. Cellular excitability and spine number and density of Thy1-COP4-expressing PrL pyramidal neurons were analyzed. Furthermore, in the same samples in which PrL pyramidal neurons were electrophysiologically recorded, Thy1-COP4-expressing BLA pyramidal neurons were sorted to purify individual cell-specific RNA for transcriptomic analyses. Transcriptome-wide analyses were carried out by RNA-sequencing, after total RNA ultra-low input library preparation and sequencing in PE75 mode on Illumina platform.

To control for the effects of learned but not yet extinguished fear on cellular excitability and spinogenesis of Thy1-COP4-expressing PrL pyramidal neurons, other Thy1-COP4 mice (No-EX group, *n* = 6) were unilaterally implanted with a guide cannula on PrL sub-region of right mPFC and then fear-conditioned by using the CFC paradigm without the extinction protocol. In fact, the day after the Conditioning phase (day 2) the animals were sacrificed and cellular excitability and spine number and density of PrL pyramidal neurons were analyzed.

### 4.3. Stereotaxic Surgery and Fiber Optic Implantations

All mice were anesthetized by using Zoletil 100 (tiletamine HCl 50 mg/mL + zolazepam HCl 50 mg/mL; Virbac, Milan, Italy) and Rompun 20 (xylazine 20 mg/mL; Bayer S.p.A, Leverkusen, Germany) dissolved in a volume of saline of 4.1 mg/mL and 1.6 mg/mL, respectively and intraperitoneally injected in a volume of 7.3 mL/kg. Mice were mounted onto a stereotaxic frame (David Kopf Instruments, Tujunga, CA, USA) equipped with a mouse adapter and unilaterally implanted with optic fiber (ThorLabs, Newton, NJ, USA) above the PrL part of the right mPFC (AP: +1.8 mm, ML: +0.25 mm, DV: −2.00 mm). The coordinates from bregma were measured according to the atlas of Franklin and Paxinos (1997) and Mouse Brain Atlases (The Mouse Brain Library; www.nervenet.org). Ferrule-terminated implanted optical fibers were secured to the skull using dental acrylic.

Mice were allowed to recover from surgery for 1 week before behavioral testing. During the recovery period, they were habituated to handling and connection of the optic fiber with the optogenetic ferrule. Locations of implanted optical fibers were validated using histology in all experimental mice.

### 4.4. CFC and In Vivo Optogenetic Stimulations of the PrL Pyramidal Neurons

As previously described [71,72,73], the CFC was carried out in a soundproof conditioning chamber (50 cm long, 24.5 cm wide, 26.5 cm high) (Ugo Basile, Varese, Italy) made of gray Perspex with a metal grid floor. A video camera placed above the conditioning chamber allowed observing animal behavior. Before the behavioral testing, the mice were handled to connect the optic fiber with the optogenetic ferrule.

As depicted in Figure 1A, on day 1 (Conditioning phase), each mouse was allowed to explore the conditioning chamber for the first 3 min (Baseline). Afterward, only a sample (*n* = 26) received three foot-shocks (0.5 mA, 2.0 s, 1 min inter-shock interval), representing the US. The fear-conditioned animals (*n* = 26) were removed from the conditioning chamber after 1 min from the third foot-shock, while the not fear-conditioned animals (*n* = 20) were removed after 6 min from the insertion in the chamber, to return to their home cages. The employed foot-shock parameters evoked signs of discomfort as freezing, flinching, jumping, and vocalizing.

According to previous reports [71,72,73] on days 2, 3, 4, 7, and 14 (Extinction phase), a sample of fear-conditioned (*n* = 20) and all not fear-conditioned (*n* = 20) mice were placed again in the conditioning chamber for 6 min (Figure 1A). During the Extinction phase, no shock was delivered. From 3^rd^ min onwards of each day of Extinction phase the mice received three optogenetic or sham stimulations on PrL pyramidal neurons of mPFC, by connecting the optic fiber to a light power source (473 nm; pE2, CoolLED, Andover, UK). Light stimulation parameters were 2s, 20 Hz, 15 ms pulses, 1 pulse every minute, density 14.32–15.91 mW/mm^2^ [74]. No light was delivered on sham-stimulated mice. Notably, in vivo optogenetic manipulation of PrL pyramidal neurons was delivered in fear-conditioned mice to maintain fear memories.

The remaining fear-conditioned mice (*n* = 6) on days 2 were placed again in the conditioning chamber for 6 min (without receiving any optogenetic stimulation) and then sacrificed.

Freezing was recorded by an experimenter blind to the group the animal belonged to and freezing times during the first 3 min for Conditioning (Baseline) and Extinction phases as well as during the entire 6 min of day 14 of Extinction phase were compared among groups.

### 4.5. Slice Preparation and Electrophysiological Recordings of PrL Pyramidal Neurons

A sample of mice was anesthetized with an overdose of halothane (Sigma-Aldrich, St. Loui, MO, USA) and decapitated on day 2 (*n* = 3) or day 14 of Extinction phase (*n* = 5/group). Once removed, the brain was attached with cyanoacrylate glue to a tray and then sectioned into 275 µM-thick coronal slices by means of a vibratome (Leica VT1200s, Wetzlar, Germany). During slicing, brain was maintained in ice-cold artificial cerebrospinal fluid solution (ACSF) containing (in mM): NaCl (126), NaHCO_3_ (26), KCl (2.5), NaH_2_PO_4_ (1.25), MgSO_4_ (2), CaCl_2_ (2) and glucose (10), gassed with 95% O_2_–5% CO_2_ (pH 7.4, 300 mOsm). Slices were maintained in the same solution at room temperature (~22 °C) for at least 30 min. Then, a single slice was transferred to a recording chamber mounted on upright infrared microscopy (BX51WI Olympus, Tokyo, Japan) and continuously perfused with oxygenated ACSF (30 °C, 2.5 mL/min) for electrophysiological recordings. Cells were visualized with a 40x water-immersion objective (LumpPlanFI, Olympus, Tokyo, Japan) and by an infrared camera EM-CCD camera (ImagEm, Hamamatsu, Hamamatsu City, Japan). Patch-clamp recordings were made by borosilicate glass pipette (3–5 MΩ) pulled with a micropipette puller (P97, Sutter Instruments, Novato, CA, USA). For current-clamp experiments, the pipette was filled with a solution containing (in mM): KMeSO_4_ (140), KCL (10), HEPES (10), Mg_2_ATP (2) and Na_3_GTP (0.4) (pH adjusted to 7.25 with KOH). For voltage-clamp experiments, the intracellular solution contained (in mM): CsMeSO_3_ (140), EGTA (1), CsCl_2_, (5.5), CaCl_2_ (0.1), HEPES (1), MgCl_2_ (2) Mg-ATP (2) (pH adjusted to 7.3, 290 mOsm). Electrophysiological recordings were acquired by a Multiclamp 700b amplifier, Digidata 1550A, and pClamp 10.4 software (Molecular Devices, San Jos, CA, USA). Signals were digitized at 10 or 20 kHz and filtered at 2 kHz with a low-pass Bessel filter. For voltage-clamp experiments, series resistance was monitored by repeated 5 mV steps. Cells showing an increase of over 20% in series resistance were discarded from statistical analysis. Spontaneous synaptic events were analyzed off-line by using the Minianalysis Program (Synaptosoft Inc., Decatur, GA, USA) and ClampFit 10.2 (Molecular Devices).

### 4.6. Spine Counting of PrL Pyramidal Neurons

The remaining sample of mice was anesthetized with an overdose of Zoletil (800 mg/kg; Virbac, Milan, Italy) + Rompun (200 mg/kg; Bayer S.p.A, Leverkusen, Germany) dissolved in a volume of saline of 4.1 mg/mL and 1.6 mg/mL respectively, and intraperitoneally injected on day 2 (*n* = 3) or day 14 of Extinction phase (*n* = 5/group). The animals were decapitated, the brains were rapidly removed, fixed in 4% paraformaldehyde for 24 h, and then cryoprotected in 30% sucrose solution. The brains were cut on a freezing microtome into 50 µm-thick coronal sections. Sections were collected at the level of PrL region of mPFC (AP: from 2.68 mm to 1.80 mm from bregma) [75] and then mounted onto slides, dehydrated, and coverslipped using Fluoromount (Sigma-Aldrich).

Dendritic spine counts were performed using an optical microscope (Axio Imager M2, Zeiss, Oberkochen, Germany) equipped with a motorized stage and a camera connected to software Neurolucida 2020.1.2 (MicroBright-Field, Williston, VT, USA). Dendrites were traced with spines and images then exported to Neurolucida™ Explorer 2019.2.1 (MicroBright-Field) for spine quantitation.

Due to the difficulty of unequivocally distinguishing filopodia from long thin spines, spine counts included all types of dendritic protrusions ≤4 µm on apical dendrites regardless of their shape or actual function.

Ten dendritic segments (length 20–25 µm) were obtained for each subject of the entire sample. Spine density was calculated by measuring the length of the dendrite segment and counting the number of spines along the segment.

### 4.7. Amygdala Pyramidal Neuron-Specific RNA Sequencing

#### 4.7.1. Dissociation of Amygdala Tissue for Fluorescence-Activated Cell Sorting (FACS)

On day 14 of Extinction phase, the brains from which PrL pyramidal neurons of mPFC were electrophysiologically recorded were cut to take bilateral amygdala 1-mm punches. Manual and enzymatic dissociations were performed using the Neural Tissue Dissociation Kit (P) (Miltenyi Biotec, Bergisch Gladbach, Germany) with some modifications. Each solution was kept on ice to minimize RNA degradation. Pipette tips were pre-coated in a 0.2 µM filtered 1× PBS-0.5% BSA solution (DPBS without Mg^2+^ and Ca^2+^, Gibco by Life Technologies, Grand Island, NY, USA; BSA Fraction V (pH 7.0), PanReac AppliChem GmbH, Darmstadt, Germany). Briefly, the amygdala punches were placed on a 35 mm diameter Petri dish, cut into small pieces using a scalpel, and 1 mL of cold Hanks’ Balanced Salt Solution without Mg^2+^ and Ca^2+^ (HBBS w/o) (Sigma-Aldrich, St. Louis, MO, USA) was added. The tissue was transferred into a 1.5 mL protein LoBind tube. Additional 1 mL HBBS w/o was used to rinse the dish and added to the 1.5 mL tube. Tissue was centrifuged at 300× *g* for 2 min at room temperature, and the supernatant was carefully aspirated. Then, 975 μL of pre-heated enzyme mix 1 (enzyme P 25 μL, buffer × 950 μL) was added to the tissue, and the 1.5 mL tube was incubated for 15 min at 37 °C under slow, continuous rotation using the MACSmix Tube Rotator (Miltenyi Biotec, Bergisch Gladbach, Germany). Then, 15 μL enzyme mix 2 (enzyme A 5 μL, buffer Y 10 μL) was added to the sample. The sample was gently inverted to mix and mechanically dissociated using the wide-tipped fire-polished Pasteur pipette by pipetting up and down 10 times slowly, followed by a further incubation in the rotator for 10 min at 37 °C under slow rotation. The second round of mechanical dissociation was performed using serially fire-polished filtered-glass Pasteur pipettes with gradual diameter diminution, and pipetting slowly up and down 10 times with each pipette, or as long as until tissue pieces were not yet observable. The sample was again incubated at 37 °C for 10 min using rotator under slow rotation, before being strained through MACS Smart Strainer (70 μm) (Miltenyi Biotec, Bergisch Gladbach, Germany), placed on a 15 mL tube, pre-coated with 0.2 μM filtered 1× PBS-0.5% BSA, adding 8 mL of HBBS with Mg^2+^ and Ca^2+^. Then, the cell sample was centrifuged at 300× *g* for 10 min at room temperature and the supernatant was completely aspirated and collected into a new 15 mL tube, and centrifuged again at 300× *g* for 10 min at room temperature. The supernatant was again completely aspirated. The pellets obtained from these two centrifugations were pooled into a 1× PBS-0.5% BSA pre-coated SNAP-cap tube containing 1 mL of PBS. Finally, 20U Superase-Inhibitor (Ambion, Invitrogen, ThermoFisher Scientific, Walthem, MA, USA) was added and samples were stored on ice up to sorting.

#### 4.7.2. Cell Sorting and Isolation of Purified Pyramidal Neurons

**Figure 8:**
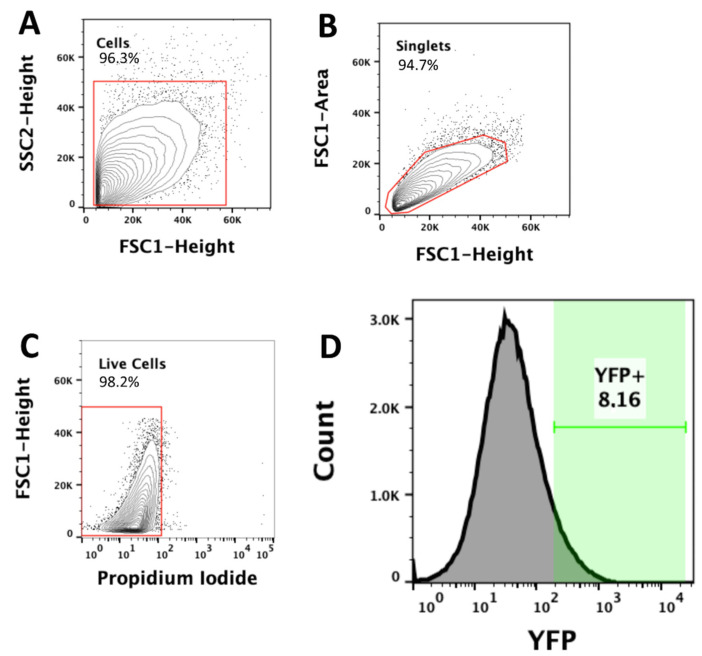
Gating strategy for amygdala pyramidal neurons cell purification. Amygdala pyramidal neurons, excited with a ~488 nm blue laser, were sorted on the basis of their physical parameters (forward scatter, FSC, and side scatter, SSC, light scattering) (**A**), singlets (**B**), propidium iodide negative (live cells) (**C**) and Yellow Fluorescent Protein (YFP) intensity (**D**), and the positive cells were collected. Cells are sorted by high-speed cell sorting (Moflo Astrios EQ).

For the instrument set-up, the samples collected from the amygdala of wild-type YFP-negative mice were used to gate YFP-positive neurons based on forward scatter (FSC) and side scatter (SSC) light scattering and to set YFP negativity. Afterwards, amygdala samples were collected from the Thy1-COP4 mice and stained with 1 μL of propidium iodide (PI) in order to identify dead cells. Pyramidal neurons were then sorted by using the MoFlo Astrios EQ (Beckman Coulter, Brea, CA, USA) and the pyramidal neurons characterized by YFP were collected on the basis of their physical parameters, singlets, PI negative (live cells), and YFP intensity (Figure 8). For initial characterization, samples were collected in PBS and samples examined under a fluorescent microscope to verify correct sorting. Thereafter, cells were sorted directly into ice-cold lysis buffer (Reliaprep RNA Cell Miniprep System, Promega, Fitchburg, WI, USA), mixed by vortexing, kept on ice, and then stored at −80 °C until RNA extraction.

#### 4.7.3. RNA-Seq Library Preparation

After thawing on ice in presence of additional proteinase K, RNA was isolated according to manufacturer’s instructions including on-column DNase treatment. RNA samples were quantified and the quality was tested by Agilent 2100 Bioanalyzer RNA assay (Agilent Technologies, Santa Clara, CA, USA) or Caliper (PerkinElmer, Waltham, MA, USA) (Table 5).

Library preparation and sequencing were performed at IGATechnology (Udine, Italy). At least 3 independent biological replicates were used for each group (Table 5). Each replicate corresponds to the amygdala of a single Thy1-COP4 animal.

Libraries were generated from each sample individually, starting from 0.05–1.4 ng of total RNA, using the Ovation SoLo RNA-seq kit for Ultra-low input (NuGEN, Tecan Genomics, Redwood City, CA, USA), following the manufacturer’s instructions (library type: fr-second strand). Final libraries were checked with both Qubit 2.0 Fluorometer (Invitrogen by Life technologies, Carlsbad, CA, USA) and Agilent Bioanalyzer DNA assay (Agilent Technologies, Santa Clara, CA, USA) or Caliper (PerkinElmer, Waltham, MA, USA). Libraries were then prepared for sequencing and sequenced on paired-end 2 × 75 bp mode on NextSeq500 (Illumina, San Diego, CA, USA) producing 35.2 MR on average (min 30.4 MR, max 51.7 MR). For the processing of raw data (format conversion and de-multiplexing), Bcl2Fastq 2.20 version of the Illumina pipeline was used. Sequencing data have been deposited in the NCBI Short Read Archive (https://www.ncbi.nlm.nih.gov/geo; GEO accession number GSE162417).

#### 4.7.4. Analysis of RNA Sequencing Data

GRCm38.p6 genome was used to map the reads, and transcript abundances were estimated using Salmon v1.2 [76]. To obtain gene-level count matrices the quantification data were imported using tximport [77]. All further analyses based on these count matrices were performed with the free software R v4.0.2, Bioconductor v3.11 [78], and the package NOISeq v2.31.0 [79]. Differences in RNA composition between samples were corrected by the Trimmed mean of M-values (TMM) normalization [80], and filtered for low counts based on a count per million reads (CPM) criteria. Subsequently, ARSyNseq was used to remove the technical batch effect and NOISeqBIO was used to assess differential gene expression (*q* > 0.95, equivalent to FDR-corrected *p* < 0.05) [79,81]. GO and KEGG pathway analyses were performed by using ClusterProfiler v3.16.1 [82]. The enrichment map method was used to identify functional modules of mutually overlapping gene sets [82,83].

### 4.8. Statistical Analysis

As regard the behavioral results, a three-factor ANOVA (stimulation × fear × day) or a two-factor ANOVA (group × day) on freezing behavior (measured during 0–3 min of contextual FC) and one-factor ANOVA on freezing behavior (measured during 0–6 min of day 14) were used. Newman-Keuls post-hoc comparisons were applied when permitted.

As regard the morphological results, one-factor ANOVAs on spine number and density were used. Newman-Keuls post-hoc comparisons were applied when permitted.

As regard the electrophysiological results, Pearson r Correlation test (for evoked firing activities and EPSC values) and Mann-Whitney U Test (for rheobase and EPSC values) were used.

As for the behavioral, morphological, and electrophysiological results, all analyses were performed by using Statistica 7.0 for Windows (TIBCO Software Inc., Aliso Viejo, CA, USA) and values of *p* = 0.05 were considered statistically significant.

As regard the transcriptomic results, after TMM normalization and low counts filtering, the resulting genes underwent the downstream analysis. Batch effect correction was applied with ARSyN and a PCA was performed to assess sample clustering based on their expression profiles. Differential expression analysis was performed on Group × Condition design and DEGs were identified using NOISeqBIO, a non-parametric analysis for biological replicates. Significant differentially expressed genes were identified for a *q* > 0.95, equivalent to an FDR-corrected *p* < 0.05. Subsequently, GO and KEGG over-representation analyses were performed and significant pathways were represented by means of enrichment map method to visualize and interpret results.

## 5. Conclusions

Given the critical role of the pyramidal neurons of amygdala and PrL cortex in fear processing, the characterization of the structural, neurophysiological, and molecular changes of this neuronal population associated to adaptive or maladaptive fear extinction may provide valuable insight for the study of, and therapeutic interventions in, fear-related and psychiatric disorders.

## Figures and Tables

**Table 1:** The top 20 DEGs identified by differential expression profiling from RNA extracted by amygdala pyramidal neurons. Comparison between OPTO FEAR vs. SHAM FEAR groups.

Gene Symbol	Gene Name	Fold Change (log2 Scale)	OPTO FEAR adj.mean	SHAM FEAR adj.mean
*Slc6a17*	solute carrier family 6 (neurotransmitter transporter), member 17	−4.24	12.86	242.95
*2010300C02Rik*	RIKEN cDNA 2010300C02 gene	−4.02	6.95	113.03
*Lmo3*	LIM domain only 3	−3.80	12.43	172.75
*Celf5*	CUGBP, Elav-like family member 5	−3.74	12.58	168.18
*Hap1*	huntingtin-associated protein 1	−3.65	13.22	166.13
*Papss2*	3′-phosphoadenosine 5′-phosphosulfate synthase 2	−3.60	9.79	118.38
*Sphkap*	SPHK1 interactor, AKAP domain containing	−3.52	23.12	265.56
*Ccdc148*	coiled-coil domain containing 148	−3.51	9.18	104.53
*Lrp11*	low density lipoprotein receptor-related protein 11	−3.50	8.59	97.11
*6330403K07Rik*	RIKEN cDNA 6330403K07 gene	−3.48	6.08	67.76
*Ube2ql1*	ubiquitin-conjugating enzyme E2Q family-like 1	−3.47	5.54	61.52
*Brinp1*	bone morphogenic protein/retinoic acid inducible neural specific 1	−3.47	11.69	129.49
*Arpp21*	cyclic AMP-regulated phosphoprotein, 21	−3.44	33.80	366.38
*Gabra4*	gamma-aminobutyric acid (GABA) A receptor, subunit alpha 4	−3.41	12.24	130.15
*Csmd1*	CUB and Sushi multiple domains 1	−3.39	25.79	269.68
*Kcnh3*	potassium voltage-gated channel, subfamily H (eag-related), member 3	−3.34	5.02	50.67
*Agt*	angiotensinogen (serpin peptidase inhibitor, clade A, member 8)	−3.28	33.63	327.00
*St6gal2*	beta galactoside alpha 2,6 sialyltransferase 2	−3.28	9.03	87.47
*Bcl2*	B cell leukemia/lymphoma 2	−3.27	17.48	169.00
*Cbarp*	calcium channel, voltage-dependent, beta subunit associated regulatory protein	−3.23	16.69	156.53

**Table 2:** The top 20 DEGs identified by differential expression profiling from RNA extracted by amygdala pyramidal neurons. Comparison between OPTO NOT FEAR vs. SHAM NOT FEAR groups.

Gene Symbol	Gene Name	Fold Change (log2 Scale)	OPTO NOT FEAR adj.mean	SHAM NOT FEAR adj.mean
*0610010K14Rik*	RIKEN cDNA 0610010K14 gene	−4.83	2.44	69.50
*Gm10163*	predicted pseudogene 10163	−4.15	8.60	153.17
*Dipk2a*	divergent protein kinase domain 2A	−3.72	34.80	457.61
*Nab1*	Ngfi-A binding protein 1	−3.61	30.00	366.69
*Nkap*	NFKB activating protein	−3.56	19.30	227.98
*Slc6a9*	solute carrier family 6 (neurotransmitter transporter, glycine), member 9	−3.53	67.13	778.02
*Agt*	angiotensinogen (serpin peptidase inhibitor, clade A, member 8)	−3.51	33.15	378.72
*Cpm*	carboxypeptidase M	−3.47	45.96	509.16
*Ermp1*	endoplasmic reticulum metallopeptidase 1	−3.40	61.64	652.31
*Gm15500*	predicted pseudogene 15500	−3.40	54.43	573.54
*Dazap2*	DAZ associated protein 2	−3.36	59.16	608.47
*Rtl8b*	retrotransposon Gag like 8B	−3.35	33.48	340.80
*Polm*	polymerase (DNA directed), mu	−3.32	6.50	64.73
*Gpx4*	glutathione peroxidase 4	−3.31	39.34	391.42
*Sos1*	SOS Ras/Rac guanine nucleotide exchange factor 1	−3.31	73.41	727.62
*Ctr9*	CTR9 homolog, Paf1/RNA polymerase II complex component	−3.30	67.94	667.74
*Tmem184c*	transmembrane protein 184C	−3.29	47.58	464.32
*Dzank1*	double zinc ribbon and ankyrin repeat domains 1	−3.27	36.48	352.91
*D430019H16Rik*	RIKEN cDNA D430019H16 gene	−3.27	35.99	346.81
*Klhl9*	kelch-like 9	−3.24	57.35	543.23

**Table 3:** The top 20 DEGs identified by differential expression profiling from RNA extracted by amygdala pyramidal neurons Comparison between OPTO FEAR vs. OPTO NOT FEAR groups.

Gene Symbol	Gene Name	Fold Change (log2 Scale)	OPTO FEAR adj.mean	OPTO NOT FEAR adj.mean
*Ube2ql1*	ubiquitin-conjugating enzyme E2Q family-like 1	−5.13	5.54	194.27
*Slc6a17*	solute carrier family 6 (neurotransmitter transporter), member 17	−5.12	12.86	448.21
*Papss2*	3′-phosphoadenosine 5′-phosphosulfate synthase 2	−4.57	9.79	232.51
*Hap1*	huntingtin-associated protein 1	−4.29	13.22	258.18
*Ngef*	neuronal guanine nucleotide exchange factor	−4.28	18.41	358.22
*Cpm*	carboxypeptidase M	4.24	871.36	45.96
*Thy1*	thymus cell antigen 1, theta	−4.22	88.57	1649.90
*Slc6a9*	solute carrier family 6 (neurotransmitter transporter, glycine), member 9	4.16	1202.20	67.13
*Arpp21*	cyclic AMP-regulated phosphoprotein, 21	−4.15	33.80	599.42
*Enc1*	ectodermal-neural cortex 1	−4.10	54.26	928.57
*Celf5*	CUGBP, Elav-like family member 5	−4.08	12.58	212.32
*Ermp1*	endoplasmic reticulum metallopeptidase 1	4.04	1016.54	61.64
*Ptprg*	protein tyrosine phosphatase, receptor type, G	−4.04	36.77	603.55
*Cxcl14*	chemokine (C-X-C motif) ligand 14	−4.02	19.49	315.59
*Chrna4*	cholinergic receptor, nicotinic, alpha polypeptide 4	−4.01	3.93	63.10
*Arsg*	arylsulfatase G	4.01	580.93	36.15
*Kcnq2*	potassium voltage-gated channel, subfamily Q, member 2	−4.00	27.21	436.00
*Sfxn1*	sideroflexin 1	−3.99	15.50	246.04
*Thrb*	thyroid hormone receptor beta	−3.91	13.85	208.27
*Lmo3*	LIM domain only 3	−3.89	12.43	183.70

**Table 4:** The top 20 DEGs identified by differential expression profiling from RNA extracted by amygdala pyramidal neurons. Comparison between SHAM FEAR vs. SHAM NOT FEAR groups.

Gene Symbol	Gene Name	Fold Change (log2 Scale)	SHAM FEAR adj.mean	SHAM NOT FEAR adj.mean
*Gm13574*	predicted gene 13574	−3.48	2.78	30.95
*Gm49601*	predicted gene, 49601	2.45	17.99	3.30
*4931406B18Rik*	RIKEN cDNA 4931406B18 gene	−2.31	10.68	52.82
*D430019H16Rik*	RIKEN cDNA D430019H16 gene	−2.30	23.45	115.51
*Sphkap*	SPHK1 interactor, AKAP domain containing	−2.25	73.14	346.81
*Slx4ip*	SLX4 interacting protein	1.98	265.56	67.29
*Smcr8*	Smith-Magenis syndrome chromosome region, candidate 8 homolog (human)	−1.97	21.56	84.55
*Olfr1233*	olfactory receptor 1233	1.81	403.22	115.33
*Hcn2*	hyperpolarization-activated, cyclic nucleotide-gated K+ 2	1.80	27.04	7.74
*Sumf1*	sulfatase modifying factor 1	1.79	859.93	248.23
*Cd99l2*	CD99 antigen-like 2	1.60	106.02	34.94
*Kif6*	kinesin family member 6	1.59	182.58	60.68
*Plaa*	phospholipase A2, activating protein	1.55	185.44	63.29
*Clstn3*	calsyntenin 3	1.51	344.56	120.72
*Adcyap1r1*	adenylate cyclase activating polypeptide 1 receptor 1	1.35	235.84	92.81
*Ino80*	INO80 complex subunit	−1.28	326.26	789.84
*Cpe*	carboxypeptidase E	1.27	359.63	148.70
*Uty*	ubiquitously transcribed tetratricopeptide repeat gene, Y chromosome	−1.23	2559.64	6021.69
*Vmn2r114*	vomeronasal 2, receptor 114	−1.21	448.82	1040.79
*Gm13574*	predicted gene 13,574	−1.19	720.62	1641.20

**Table 5:** Sketch of RNA samples extracted by amygdala pyramidal YFP neurons and sequenced in PE75 mode. Only samples used for sequencing, i.e., 14 over the total, are reported. The samples that did not pass quality control were excluded from further analyses.

Conditioning	PrL Stimulation	Sample Name	RNA (ng)	Library (ng)	Reads (M)
FEAR	OPTO	ID_FO1	0.540	0.270	33.954
FEAR	OPTO	ID_FO2	0.846	0.423	38.971
FEAR	OPTO	ID_FO3	0.225	0.112	40.455
FEAR	OPTO	ID_FO4	0.410	0.205	34.162
FEAR	SHAM	ID_FS1	0.140	0.070	34.804
FEAR	SHAM	ID_FS2	2.760	1.380	37.340
FEAR	SHAM	ID_FS3	0.640	0.320	30.547
NOT FEAR	OPTO	ID_NF01	1.770	0.885	30.483
NOT FEAR	OPTO	ID_NF02	0.240	0.120	32.432
NOT FEAR	OPTO	ID_NF03	0.510	0.255	34.756
NOT FEAR	SHAM	ID_NFS1	0.360	0.170	31.432
NOT FEAR	SHAM	ID_NFS2	0.520	0.260	30.438
NOT FEAR	SHAM	ID_NFS3	0.110	0.055	36.824
NOT FEAR	SHAM	ID_NFS4	1.060	0.530	54.741

## Data Availability

All data support the findings of this study are available from the corresponding author upon reasonable request.

## Supplementary Materials

The following are available online at https://www.mdpi.com/1422-0067/22/2/810/s1.
