# Supplementary material for: Optogenetic Stimulation of Prelimbic Pyramidal Neurons Maintains Fear Memories and Modulates Amygdala Pyramidal Neuron Transcriptome
Source: Int J Mol Sci. 2021 Jan 15;22(2):810. doi: 10.3390/ijms22020810 (PMC7830910; doi:10.3390/ijms22020810)
Supplement: Supplementary file 1 [file ijms-22-00810-s001.zip › Supplementary Files/Supplementary Figure 1_rev.pdf]

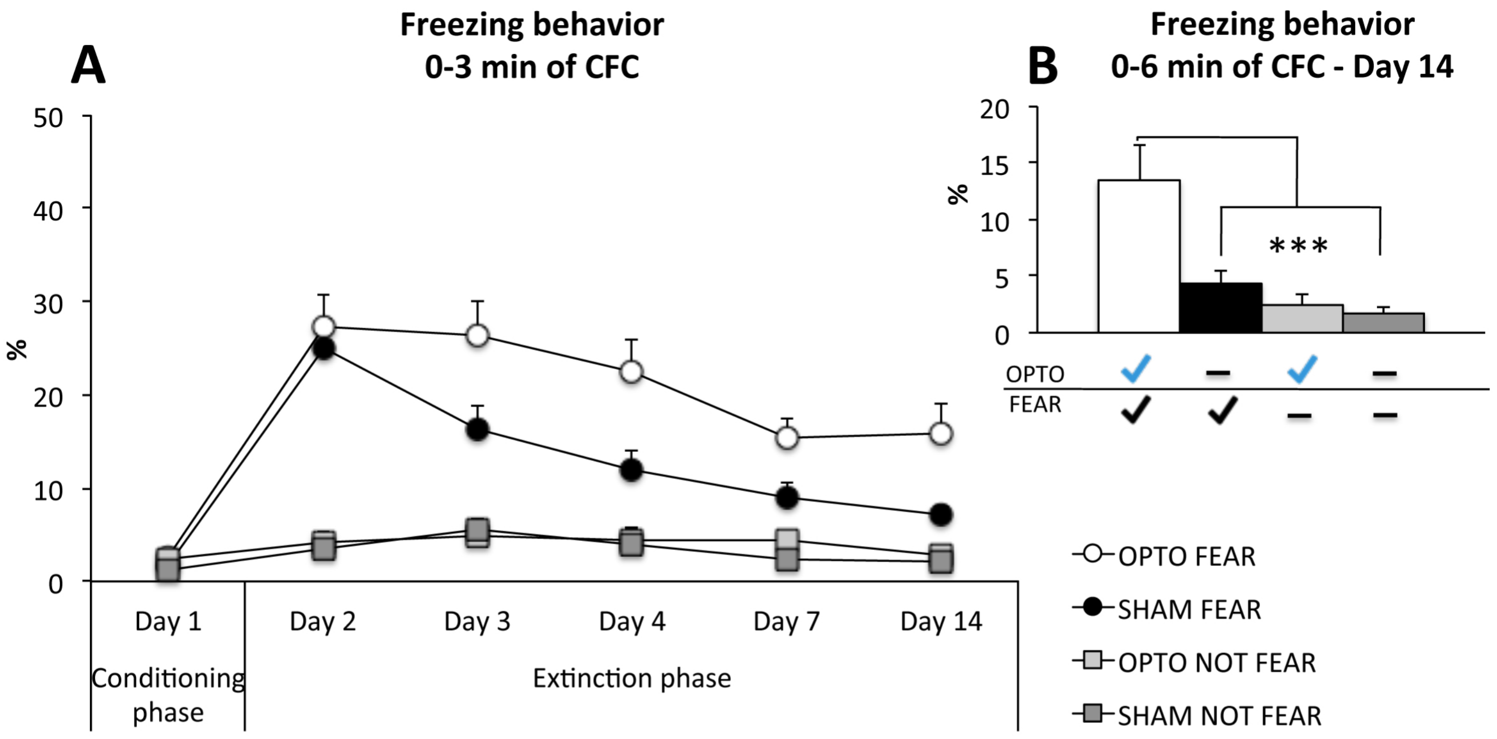

Supplementary Figure 1. Behavioral results of *in vivo* optogenetics of the PreLimbic (PrL) pyramidal neurons during Contextual Fear Conditioning (CFC). A) Percentage of freezing times measured during 0-3 min of CFC. All animals showed similar responses in the Conditioning phase and only the fear-conditioned animals (OPTO FEAR and SHAM FEAR groups) showed increased percentage of freezing times on day 2. While SHAM FEAR group progressively extinguished fear memories over time, an impaired extinction of fear memories was observed in OPTO FEAR group. B) Percentage of Freezing times measured during 0-6 min of day 14. OPTO FEAR group showed the highest percentage of freezing times in comparison to the remaining groups (*** P=0.0005).
