# Supplementary material for: Optogenetic Stimulation of Prelimbic Pyramidal Neurons Maintains Fear Memories and Modulates Amygdala Pyramidal Neuron Transcriptome
Source: Int J Mol Sci. 2021 Jan 15;22(2):810. doi: 10.3390/ijms22020810 (PMC7830910; doi:10.3390/ijms22020810)
Supplement: Supplementary file 1 [file ijms-22-00810-s001.zip › Supplementary Files/Supplementary Figure 2_Unforgettable_Laricchiuta et al. 2020.pdf]

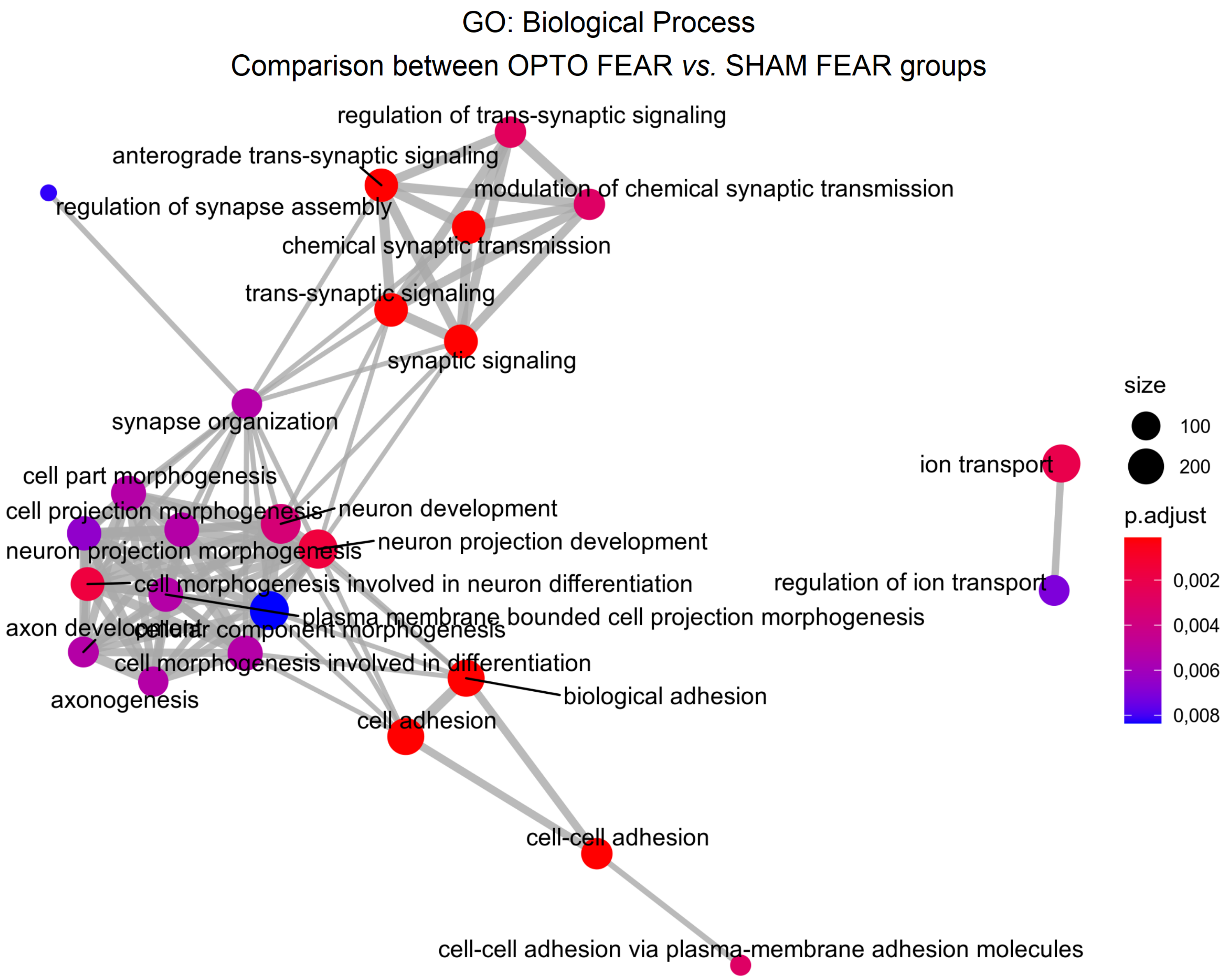
GO: Biological Process
Comparison between OPTO FEAR vs. SHAM FEAR groups
regulation of trans-synaptic signaling
anterograde trans-synaptic signaling
modulation of chemical synaptic transmission
regulation of synapse assembly
chemical synaptic transmission
trans-synaptic signaling
synaptic signaling
synapse organization
cell part morphogenesis
cell projection morphogenesis
neuron development
neuron projection morphogenesis
neuron projection development
cell morphogenesis involved in neuron differentiation
plasma membrane bounded cell projection morphogenesis
axon development
cellular component morphogenesis
cell morphogenesis involved in differentiation
biological adhesion
axonogenesis
cell adhesion
cell-cell adhesion
cell-cell adhesion via plasma-membrane adhesion molecules
ion transport
regulation of ion transport
size
100
200
p.adjust
0,002
0,004
0,006
0,008

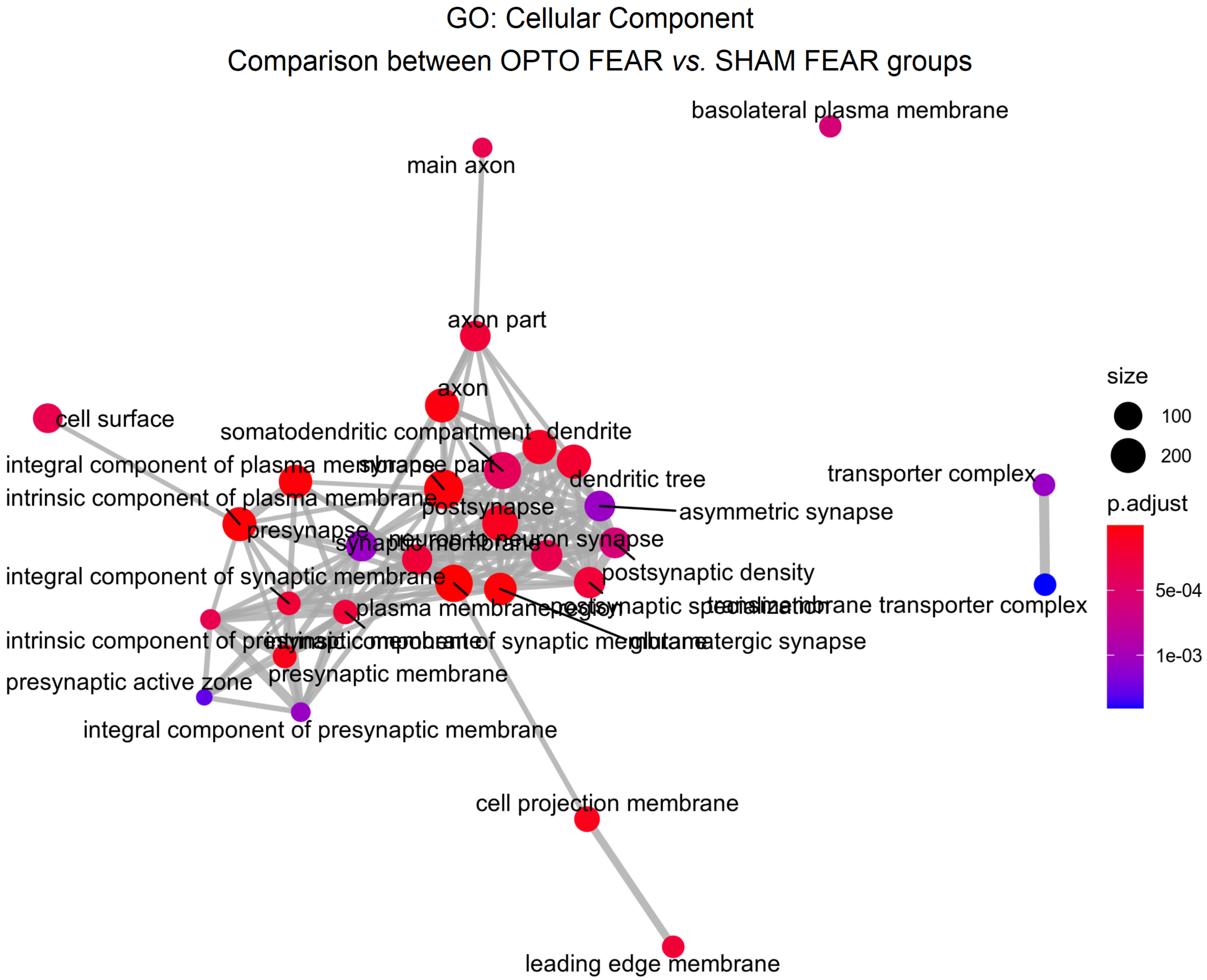
GO: Cellular Component
Comparison between OPTO FEAR *vs.* SHAM FEAR groups
basolateral plasma membrane
main axon
axon part
axon
cell surface
somatodendritic compartment
dendrite
integral component of plasma membrane
synapse part
intrinsic component of plasma membrane
dendritic tree
transporter complex
asymmetric synapse
postsynapse
presynapse
synaptic membrane
neuron to neuron synapse
postsynaptic density
integral component of synaptic membrane
plasma membrane region
postsynaptic specialization
transmembrane transporter complex
glutamatergic synapse
presynaptic membrane
presynaptic active zone
integral component of presynaptic membrane
cell projection membrane
leading edge membrane
size
100
200
p.adjust
5e-04
1e-03

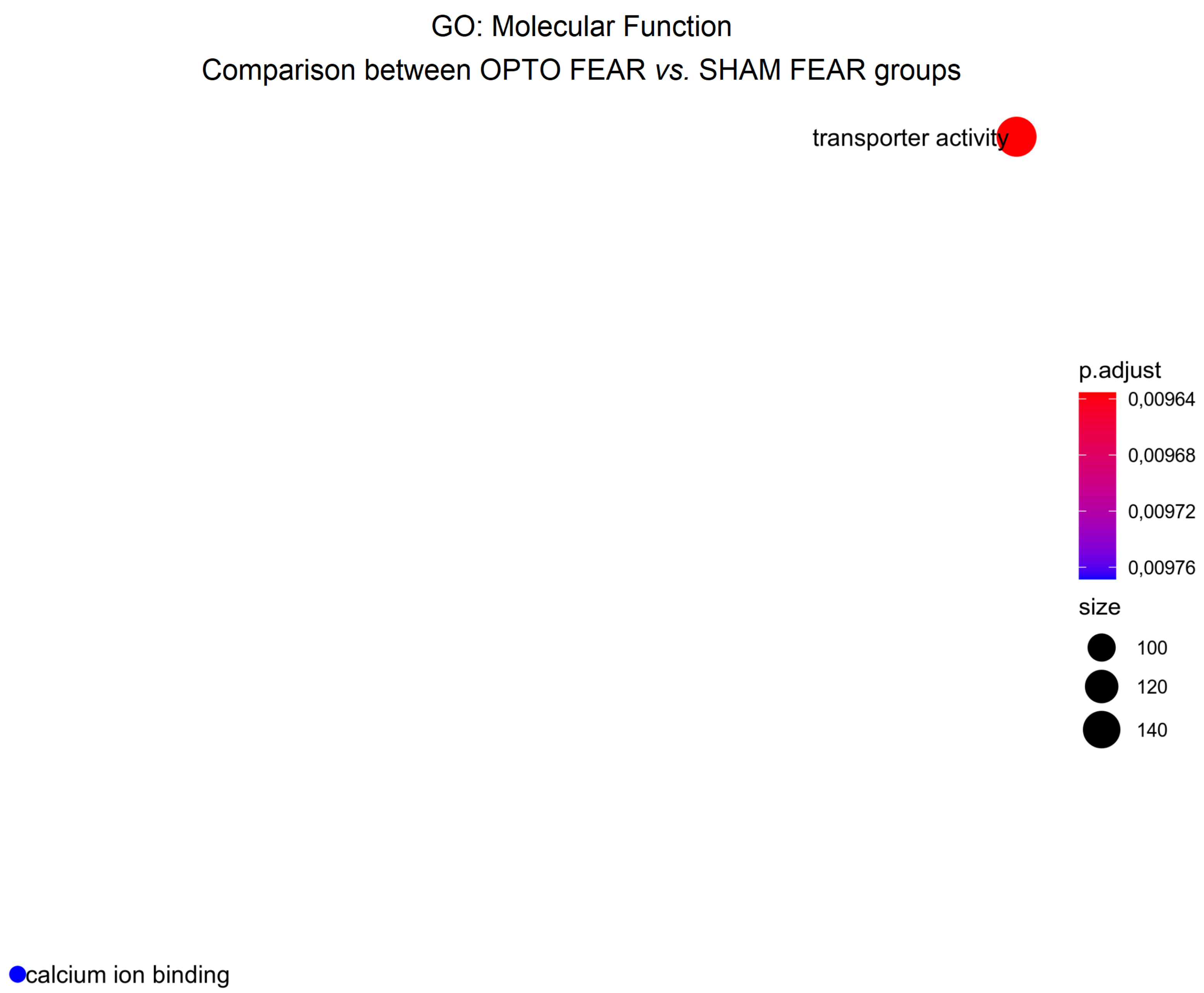
GO: Molecular Function
Comparison between OPTO FEAR vs. SHAM FEAR groups
transporter activity
calcium ion binding
p.adjust
0,00964
0,00968
0,00972
0,00976
size
100
120
140

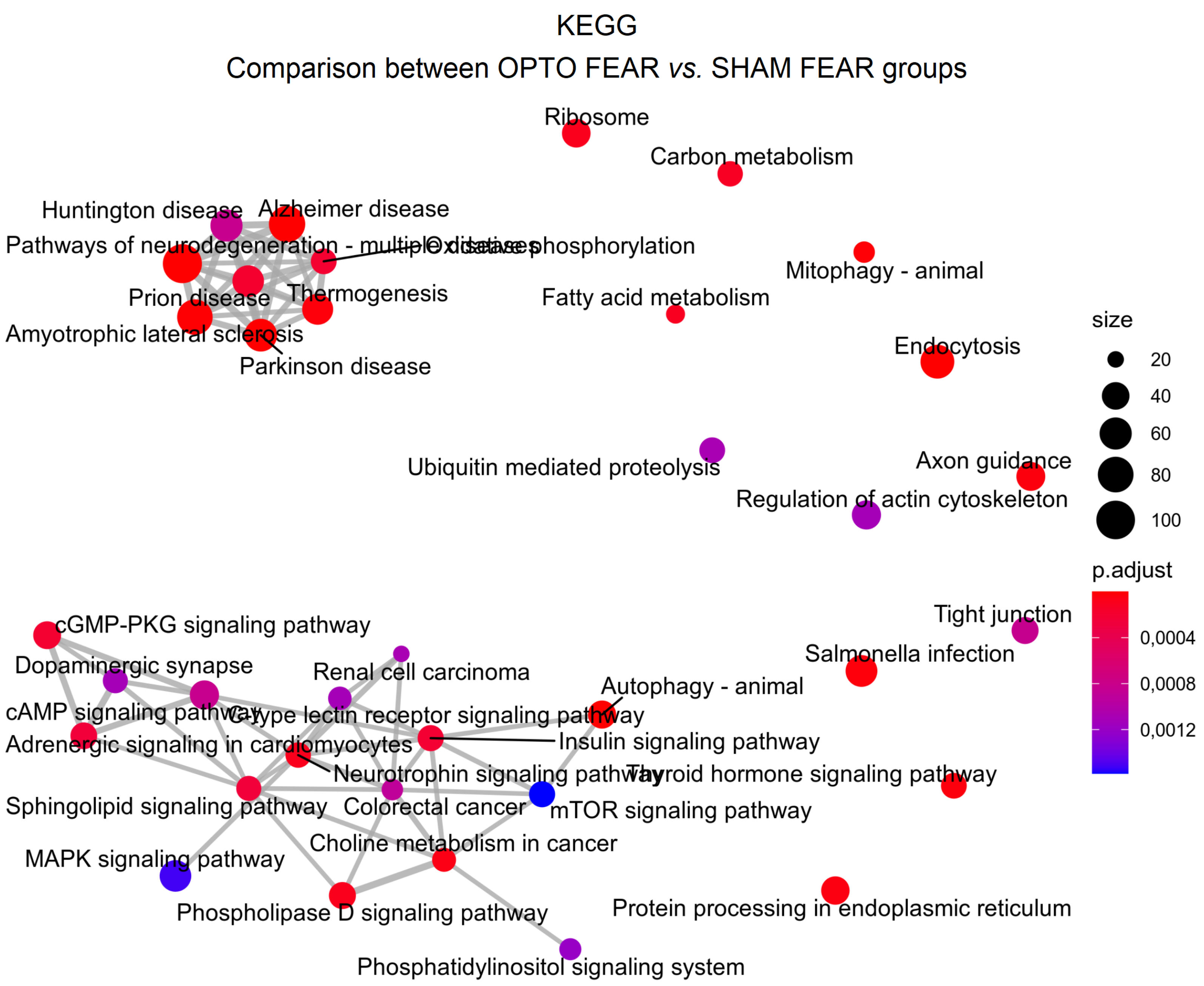
KEGG
Comparison between OPTO FEAR vs. SHAM FEAR groups
Ribosome
Carbon metabolism
Huntington disease
Alzheimer disease
Pathways of neurodegeneration - multiple diseases
Oxidative phosphorylation
Mitophagy - animal
Prion disease
Thermogenesis
Fatty acid metabolism
Amyotrophic lateral sclerosis
Parkinson disease
Endocytosis
size
20
40
60
80
100
Ubiquitin mediated proteolysis
Axon guidance
Regulation of actin cytoskeleton
p.adjust
0,0004
0,0008
0,0012
Tight junction
cGMP-PKG signaling pathway
Salmonella infection
Dopaminergic synapse
Renal cell carcinoma
Autophagy - animal
cAMP signaling pathway
C-type lectin receptor signaling pathway
Insulin signaling pathway
Adrenergic signaling in cardiomyocytes
Neurotrophin signaling pathway
Thyroid hormone signaling pathway
Sphingolipid signaling pathway
Colorectal cancer
mTOR signaling pathway
Choline metabolism in cancer
MAPK signaling pathway
Phospholipase D signaling pathway
Protein processing in endoplasmic reticulum
Phosphatidylinositol signaling system

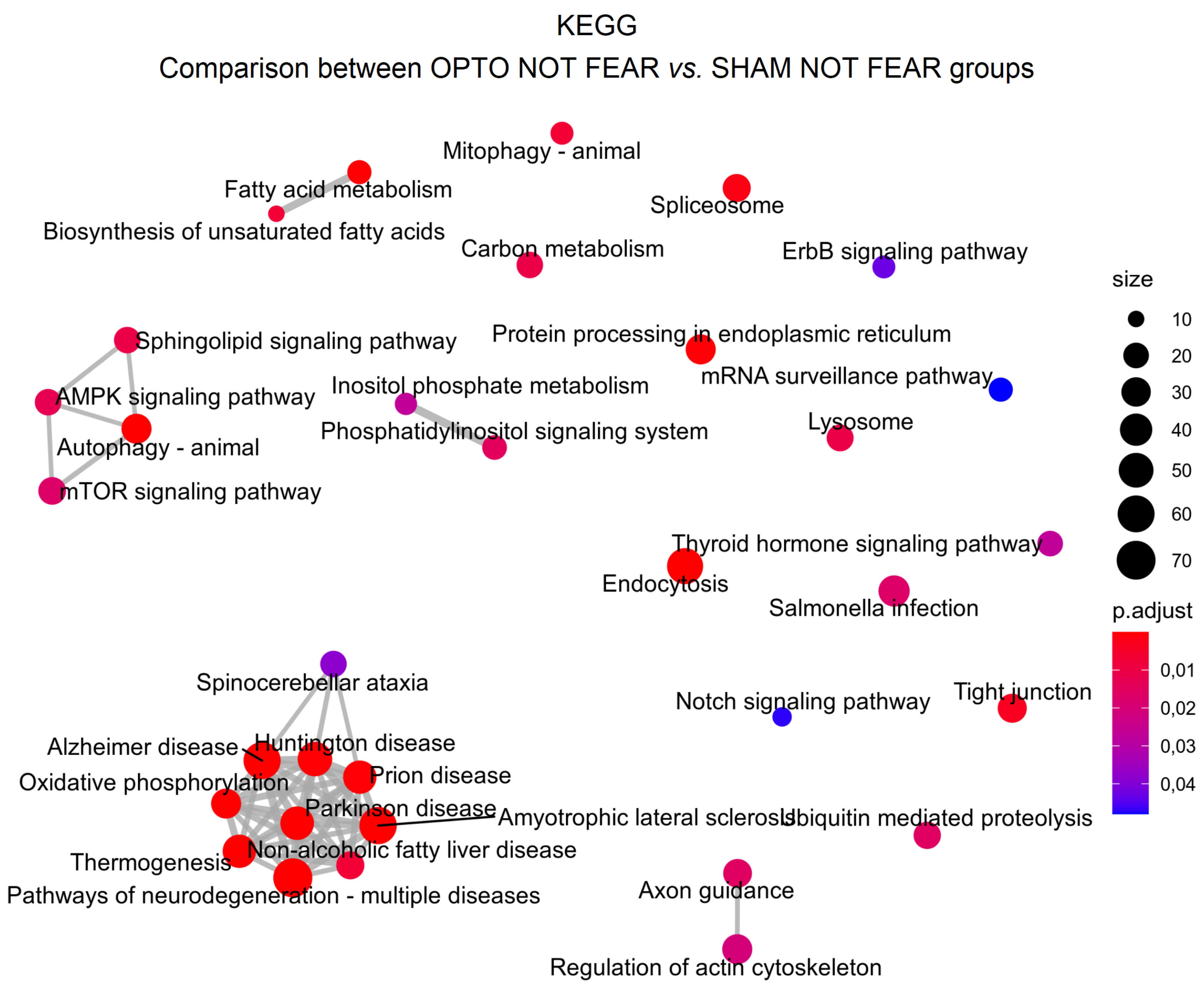
KEGG
Comparison between OPTO NOT FEAR vs. SHAM NOT FEAR groups
Mitophagy - animal
Fatty acid metabolism
Biosynthesis of unsaturated fatty acids
Spliceosome
Carbon metabolism
ErbB signaling pathway
Sphingolipid signaling pathway
Protein processing in endoplasmic reticulum
AMPK signaling pathway
Inositol phosphate metabolism
mRNA surveillance pathway
Autophagy - animal
Phosphatidylinositol signaling system
Lysosome
mTOR signaling pathway
Thyroid hormone signaling pathway
Endocytosis
Salmonella infection
Spinocerebellar ataxia
Notch signaling pathway
Tight junction
Alzheimer disease
Huntington disease
Prion disease
Oxidative phosphorylation
Parkinson disease
Amyotrophic lateral sclerosis
Ubiquitin mediated proteolysis
Non-alcoholic fatty liver disease
Thermogenesis
Pathways of neurodegeneration - multiple diseases
Axon guidance
Regulation of actin cytoskeleton
size
10
20
30
40
50
60
70
p.adjust
0,01
0,02
0,03
0,04

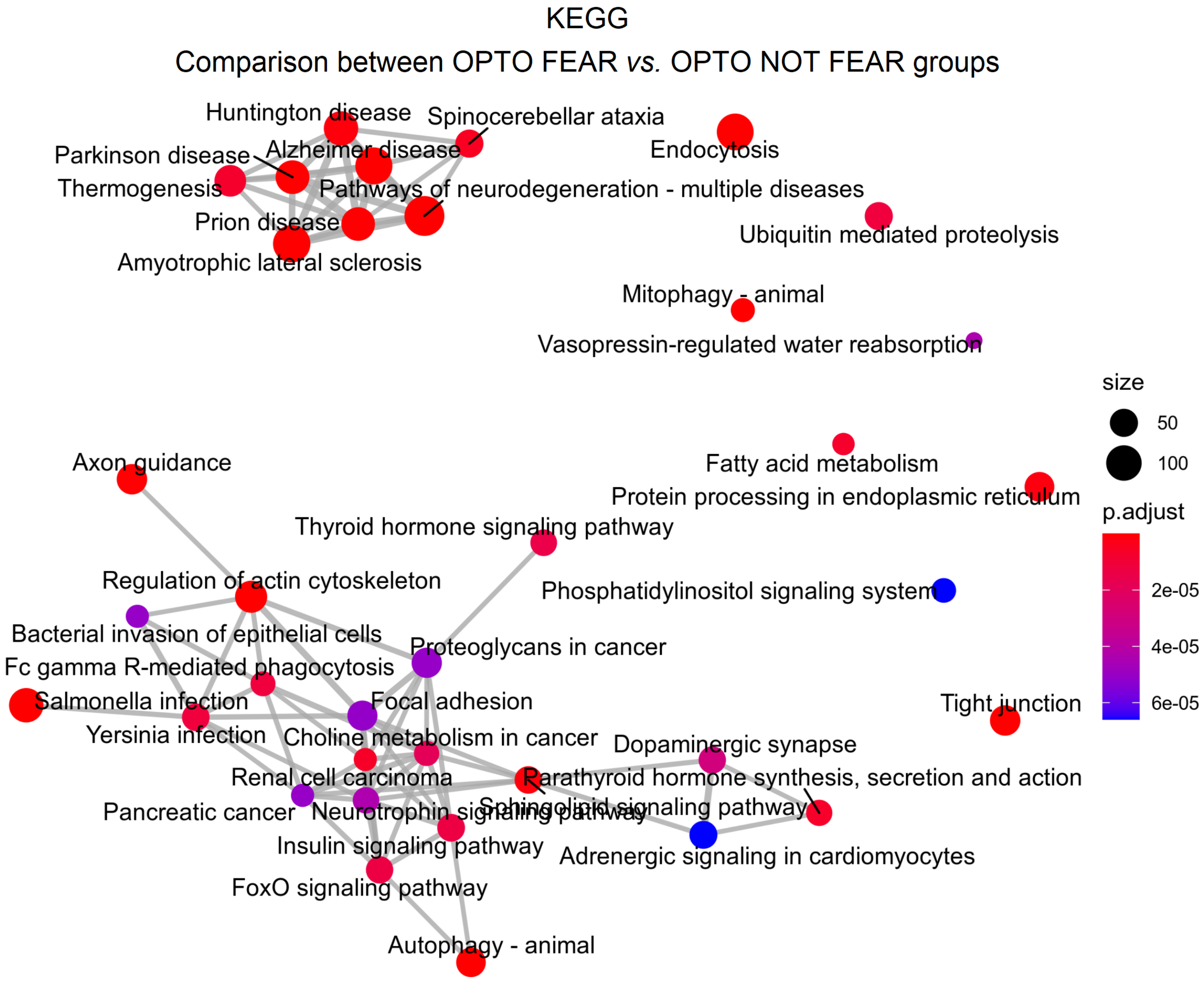

KEGG
Comparison between OPTO FEAR vs. OPTO NOT FEAR groups
Huntington disease
Spinocerebellar ataxia
Endocytosis
Parkinson disease
Alzheimer disease
Thermogenesis
Pathways of neurodegeneration - multiple diseases
Prion disease
Ubiquitin mediated proteolysis
Amyotrophic lateral sclerosis
Mitophagy - animal
Vasopressin-regulated water reabsorption
size
50
100
Axon guidance
Fatty acid metabolism
Protein processing in endoplasmic reticulum
p.adjust
Thyroid hormone signaling pathway
Regulation of actin cytoskeleton
Phosphatidylinositol signaling system
2e-05
Bacterial invasion of epithelial cells
Proteoglycans in cancer
4e-05
Fc gamma R-mediated phagocytosis
Salmonella infection
Focal adhesion
Tight junction
6e-05
Yersinia infection
Choline metabolism in cancer
Dopaminergic synapse
Renal cell carcinoma
Parathyroid hormone synthesis, secretion and action
Pancreatic cancer
Neurotrophin signaling pathway
Sphingolipid signaling pathway
Insulin signaling pathway
Adrenergic signaling in cardiomyocytes
FoxO signaling pathway
Autophagy - animal

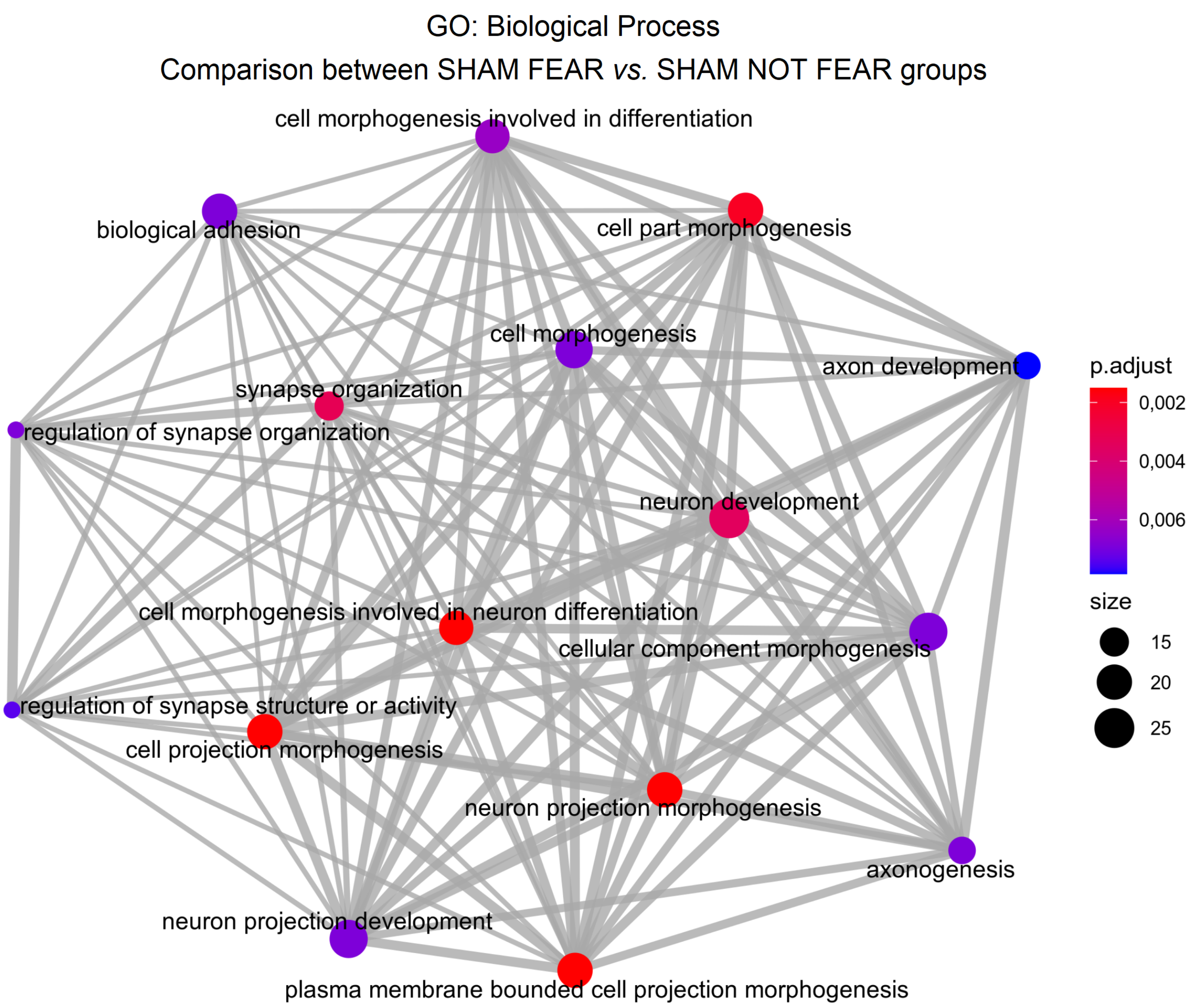
GO: Biological Process
Comparison between SHAM FEAR vs. SHAM NOT FEAR groups
cell morphogenesis involved in differentiation
biological adhesion
cell part morphogenesis
cell morphogenesis
axon development
synapse organization
regulation of synapse organization
neuron development
cell morphogenesis involved in neuron differentiation
cellular component morphogenesis
regulation of synapse structure or activity
cell projection morphogenesis
neuron projection morphogenesis
axonogenesis
neuron projection development
plasma membrane bounded cell projection morphogenesis
p.adjust
0,002
0,004
0,006
size
15
20
25

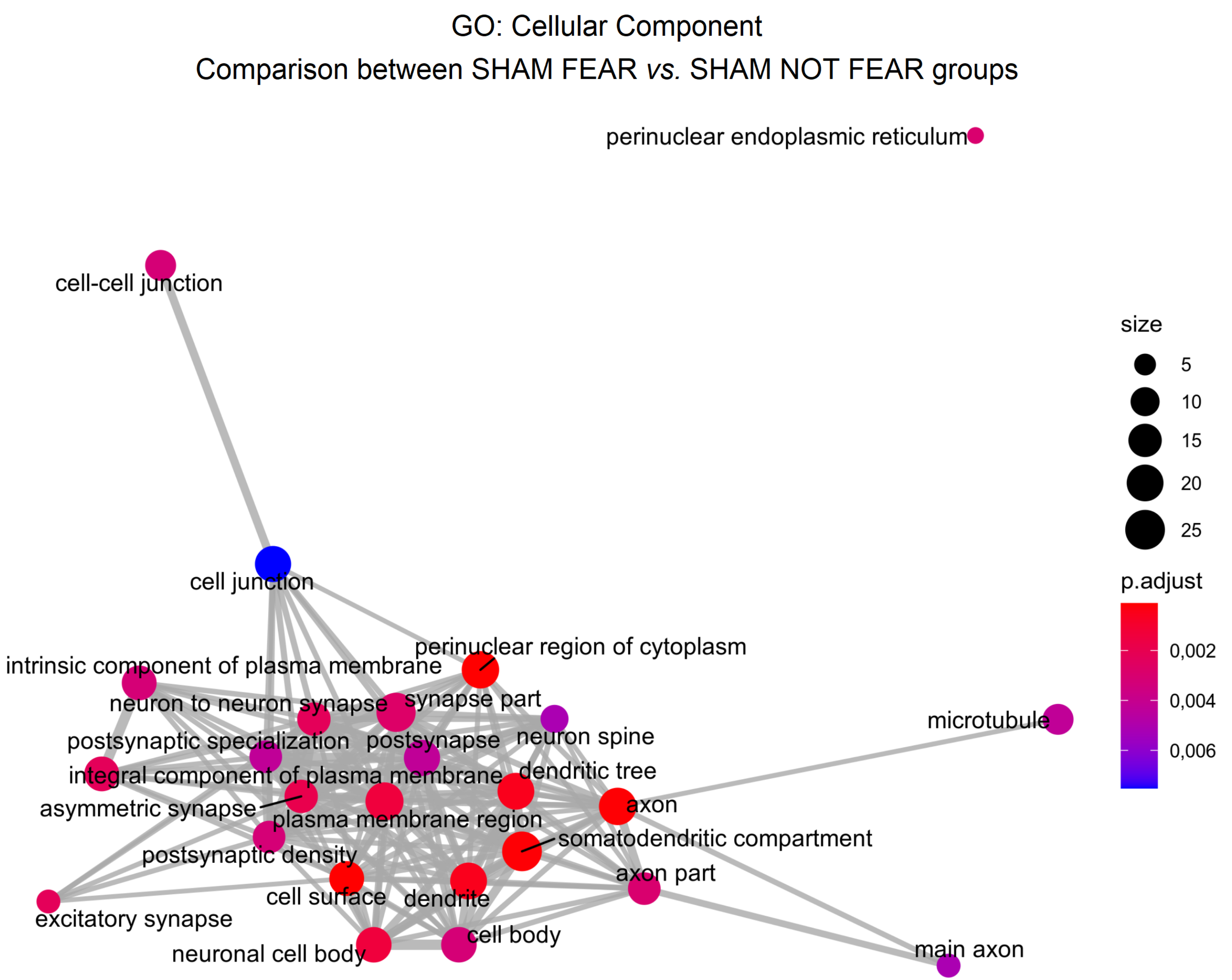
GO: Cellular Component
Comparison between SHAM FEAR vs. SHAM NOT FEAR groups
perinuclear endoplasmic reticulum
cell-cell junction
cell junction
perinuclear region of cytoplasm
intrinsic component of plasma membrane
neuron to neuron synapse
synapse part
postsynaptic specialization
postsynapse
neuron spine
integral component of plasma membrane
dendritic tree
asymmetric synapse
plasma membrane region
axon
somatodendritic compartment
postsynaptic density
axon part
cell surface
dendrite
excitatory synapse
cell body
neuronal cell body
microtubule
main axon
size
5
10
15
20
25
p.adjust
0,002
0,004
0,006

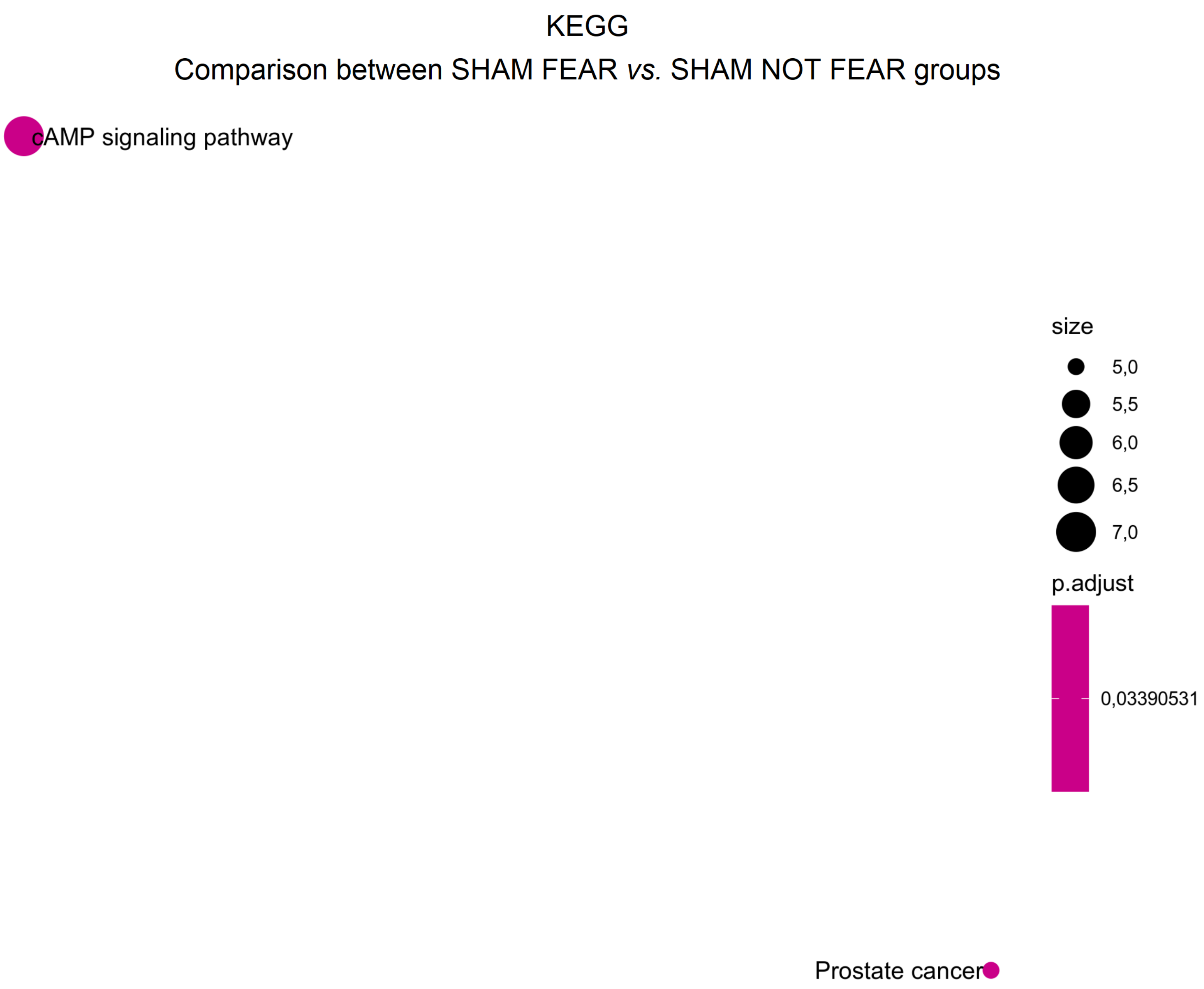

KEGG
Comparison between SHAM FEAR vs. SHAM NOT FEAR groups
cAMP signaling pathway
size
5,0
5,5
6,0
6,5
7,0
p.adjust
0,03390531
Prostate cancer
